# The power of regional heritability analysis for rare and common variant detection: simulations and application to eye biometrical traits

**DOI:** 10.3389/fgene.2013.00232

**Published:** 2013-11-19

**Authors:** Yoshinobu Uemoto, Ricardo Pong-Wong, Pau Navarro, Veronique Vitart, Caroline Hayward, James F. Wilson, Igor Rudan, Harry Campbell, Nicholas D. Hastie, Alan F. Wright, Chris S. Haley

**Affiliations:** ^1^Roslin Institute and Royal (Dick) School of Veterinary Studies, University of EdinburghMidlothian, UK; ^2^National Livestock Breeding CenterFukushima, Japan; ^3^MRC Human Genetics Unit, MRC Institute of Genetics and Molecular Medicine, University of EdinburghEdinburgh, UK; ^4^Centre for Population Health Sciences, University of EdinburghEdinburgh, UK

**Keywords:** common and rare variants, GWAS, regional heritability mapping, multiple independent effects, missing heritability

## Abstract

Genome-wide association studies (GWAS) have provided valuable insights into the genetic basis of complex traits. However, they have explained relatively little trait heritability. Recently, we proposed a new analytical approach called regional heritability mapping (RHM) that captures more of the missing genetic variation. This method is applicable both to related and unrelated populations. Here, we demonstrate the power of RHM in comparison with single-SNP GWAS and gene-based association approaches under a wide range of scenarios with variable numbers of quantitative trait loci (QTL) with common and rare causal variants in a narrow genomic region. Simulations based on real genotype data were performed to assess power to capture QTL variance, and we demonstrate that RHM has greater power to detect rare variants and/or multiple alleles in a region than other approaches. In addition, we show that RHM can capture more accurately the QTL variance, when it is caused by multiple independent effects and/or rare variants. We applied RHM to analyze three biometrical eye traits for which single-SNP GWAS have been published or performed to evaluate the effectiveness of this method in real data analysis and detected some additional loci which were not detected by other GWAS methods. RHM has the potential to explain some of missing heritability by capturing variance caused by QTL with low MAF and multiple independent QTL in a region, not captured by other GWAS methods. RHM analyses can be implemented using the software REACTA (http://www.epcc.ed.ac.uk/projects-portfolio/reacta).

## Introduction

Genome-wide association studies (GWAS) have provided valuable insights into the genetic basis of complex traits. However, the reported SNPs associated with a trait typically explain only a small proportion of genetic variance. For example, the heritability of human height is about 80% (Visscher et al., 2008), but the SNPs significantly associated with height explain only 10% of the phenotypic variance (Lango Allen et al., 2010). This has been called the “missing heritability” problem (Maher, 2008). Recently, Yang et al. (2010) showed that 45% of the phenotypic variance for human height is accounted for by common SNPs, and the difference between 10 and 45% was due to many SNPs with small effects that fail to reach significance in GWAS. Yang et al. (2010) suggested that the remaining variance evaded capture due to imperfect linkage disequilibrium (LD) between the genotyped SNPs and causal variants. Causal variants may have lower minor allele frequency (MAF) than genotyped SNPs if they are subject to purifying natural selection. In this case the variation explained by the genotyped SNPs will be lower than that due to causal variants because of low LD. A pressing need is analytical approaches adapted to capturing genetic variation due to causal variants with low MAF.

Recent studies have shown that multiple independent loci with different allele frequencies and effects are often located on the same gene region or narrow segment region. For example, seven independent alleles at 8q24 region affect prostate cancer (Haiman et al., 2007), three at the IRF5 gene affect systemic lupus erythematosus (Graham et al., 2007), and two at the IL23R gene affect Crohn's disease (Duerr et al., 2006). Such loci may escape detection by single SNP analyses if the individual allele effects are not large enough to be detected even though the cumulative effect of the whole locus on trait variance is quite large. An alternative method to analyze GWAS data is to consider an association between a trait and a composite *P*-value generated by all markers within a segment of the genome or a gene region, as opposed to individual SNPs. For a gene region, this method is called gene-based association (Neale and Sham, 2004), and can potentially increase the power to identify a causal gene that harbors several functional alleles. A new variance component approach called regional heritability mapping (RHM) that screens the genome by analyzing small regions has been suggested to capture more of the missing genetic variation (Nagamine et al., 2012). In RHM, a mixed model framework based on restricted maximum likelihood (REML) is used, and two variance components, one contributed by the whole genome and a second one by a specific genomic region, are fitted in the model to estimate genomic and regional heritabilities, respectively. RHM facilitates the capture of genetic variation that is associated with each segment of the genome by combining the effects of both common and rare variants in a region. By analyzing real data, Nagamine et al. showed that the results of RHM are correlated with results from GWAS but capture more of the missing genetic variation and identify additional quantitative trait loci (QTL).

The objective of this study is to investigate the effectiveness of RHM to capture QTL variance that is potentially not detected by single-SNP GWAS and gene-based association analyses. Such variance may be due to low MAF alleles and multiple independent QTL with small effects located on a narrow genomic region. We investigated the power to detect significant regions and accuracy of estimating regional heritability using simulation based on real genotype data from a human population. We used imputation to generate a dense map of SNPs from which to randomly select subsets at different frequencies to represent causative variants (QTL) in our simulations, using only the genotyped SNPs in the analyses. We studied the impact of different window sizes in RHM on its power and accuracy, and compared them to those of other methods that include single-SNP GWAS and a range of gene-based association approaches under several different scenarios. In addition, we also applied a RHM to analyze three eye traits to evaluate the effectiveness of this method in real data analysis.

## Materials and methods

### Population and SNP array information used in the simulation study

Samples were available from two Croatian cohorts recruited from two Dalmatian islands, Vis and Korcula, and both cohorts were approved by the Ethical Committee of the Medical School, University of Zagreb and the Multi-Centre Research Ethics Committee for Scotland. All participants gave written informed consent. The cohorts are usually referred to as CROATIA-Vis and CROATIA-Korcula, but will be referred to as Vis and Korcula in the remaining of the manuscript.

DNA samples were genotyped using the Illumina Human Hap300 (282,415 autosomal SNPs) for Vis and Illumina CNV370 (302,507 autosomal SNPs) for Korcula. Our quality control protocol excluded SNPs with MAF <0.0005, call rate <0.98 or Hardy–Weinberg equilibrium (HWE) *P*-value < 1.0 × 10^−6^. The exclusion criterion for individuals was call rate <0.97. A total of 269,706 SNPs on autosomal chromosomes were common to Vis and Korcula samples and were used in this study. In total, 953 individuals passed all quality control thresholds from Vis and 898 from Korcula, and the total of 1851 individuals were then used in the simulation study.

### SNP imputation for simulation analysis

SNPs were imputed to provide a dense map from which to select simulated causative variants (QTL). SNP imputation was performed using the IMPUTE2 program (Howie et al., 2009), incorporating 1000 Genomes Phase I (interim) data as reference panel for the Vis and Korcula genotypes, respectively. This imputation yielded posterior probabilities for genotypes at ~35 million SNPs, and an estimate of imputation quality [IMPUTE2-info score ranging from 0 (low confidence) to 1 (high confidence)]. Imputed SNPs were assigned to one of two groups depending on their IMPUTE2-info score (high_info group: 0.7 ≤ IMPUTE2-info score ≤ 1.0 and low_info group: 0.0 ≤ IMPUTE2-info score ≤ 0.5) in both populations. IMPUTE2 gives posterior probabilities for all three genotypes at each locus for each individual. Individual genotypes at each imputed SNP locus were randomly assigned according to the posterior probabilities for the three genotypes from IMPUTE2. These imputed SNPs were then assessed by the exclusion criteria of very rare MAF <0.0005 and HWE *P*-value < 1.0 × 10^−6^. The total number of selected SNPs were 3,793,540 SNPs in the low_info group and 6,704,137 SNPs in the high_info group. Comparison of imputed SNPs in the high_info group with the same SNPs genotyped on a commercial exome array indicates that the LD structure of the real population is well-represented by the imputed SNPs (see Figure S1 in Supplementary Material).

### Generating phenotypes under the null hypothesis

We simulated phenotypes under the null hypothesis based on the observed genotype data of 1851 individuals at 269,706 SNPs. The phenotypes under the null hypothesis were simulated using a polygenic model in which all SNPs were assumed to have a very small effect on the phenotype. The polygenic model was $y_{i}=\sum_{j}^{n}x_{ij}b_{j}+e_{i}$, where *x_ij_* is the genotype for *j*-th causal variant of the *i*-th individual (coded as 1, 2, or 3), *b_j_* is the allele effect of the *j*-th causal variant generated from *N*(0, 1), and *e*_*i*_ is the residual effect generated from *N*(0, σ^2^_*g*_(1/*h*^2^ − 1)). σ^2^_*g*_ is the total genetic variance of $\sum_{j}^{n}x_{ij}b_{j}$ and *h*^2^ is the setting value of genome heritability. Three setting values of genome heritability (*h*^2^ = 0.20, 0.40, and 0.80) were used for generating phenotypes. These generated phenotypes were under the null hypothesis of no phenotype-window correlation (i.e., there was no significant effect for RHM), and were then used for simulation analysis (see the details in Figure S2 in Supplementary Material).

### Simulation design and analyses

The genotyped and imputed SNPs were assigned to genomic regions on the basis of their location in 1000 Genomes Phase I (interim) information. Once the polygenic background was simulated based on the genotyped SNPs as described above, we added to it regional effects, based on the simulated genotypes at imputed SNPs. For imputed SNPs, two MAF categories were defined as low MAF (MAF < 0.10) and high MAF (MAF ≥ 0.10), respectively. We carried out simulations in the high_info and low_info imputed SNPs categories. The parameters considered in the simulation are summarized in Table 1, and shown in detail below.

**Table 1:** **Settings criteria in the simulation study**.

**Condition**	**Criteria**	
	**Low_info group**	**High_info group**
Minor allele frequency	Low MAF (0.0005 < MAF < 0.10)	Low MAF (0.0005 < MAF < 0.10), high MAF (0.10 ≤ MAF ≤ 0.50)
Number of QTL	1, 5, 10	1, 5, 10
QTL heritability	0.05	0.025, 0.050
Genome heritability	0.20, 0.40, 0.80	0.20, 0.40, 0.80

The division of the genome into regions was based on numbers of genotyped markers. A window containing 100 adjacent genotyped SNPs was named as win100. A total of 2686 win100 without overlap covered the autosomes and from these 300 win100s were randomly picked for the simulation analysis. Each win100 was divided equally into 10 10-SNP-windows (named as win10). One win10 was randomly selected from the six centermost win10s of a win100, and assumed as a gene region (i.e., we simulated causal variants in the chosen win10). This gene region contained at least 10 imputed SNPs with high MAF or low MAF. Of these 1, 5, or 10 with either high MAF or low MAF were randomly selected and assumed as QTL with joint heritabilities of either 0.025 or 0.05, which are based on the proportion of total genetic variance generated under the null hypothesis. The effect of these selected SNPs was generated, and then added to the phenotypic value generated under null hypothesis and an error value to generate a new phenotypic value with genome heritability (0.20, 0.40, and 0.80). Each selected SNP had an equal effect (and a randomly selected effect direction) that contributed to the total (“joint”) QTL variance. Each win100 was divided equally into 2, 5, and 10 windows, and each window with 50 SNPs (named as win50), 20 SNPs (named as win20) and 10 SNPs, respectively, was then used to calculate the power to detect QTL and estimate regional heritability in order to assess the optimum analysis window size for these simulated data. Average window length across all autosomal chromosomes was 1030.2 kbp for win100, 515.1 kbp for win50, 206.0 kbp for win20, and 103.0 kbp for win10.

A total of 18 RHM analyses were performed per 100-SNP window (1 win100, 2 win50, 5 win20, and 10 win10 analyses), and a *P*-value of win100 and the minimum *P*-values results of win50, win20, and win10 were selected in each window size. To determine the threshold value of win100, win50, win20, and win10, a Bonferroni correction was applied by using 2686 windows, 5372 windows, 13,430 windows, and 26,860 windows, respectively. The power to achieve 5% genome-wide significance was calculated as the proportion of replicates with a significant window for each window size, genomic heritability, number of QTL, IMPUTE2-info score levels, MAF, and QTL heritability. The regional heritability and minimum *P*-value were also computed in all replicates for win100, win50, win20, and win10, and the average value of estimated regional heritability in all simulation replicates was calculated for each window size.

We wanted to compare the power and estimated regional heritability of RHM and a range of single SNP or gene-based association methods. We used two single-SNP GWAS analyses based on the Genome-wide rapid association using mixed model and regression (GRAMMAR) method (Aulchenko et al., 2007) and the genome-wide efficient mixed-model association (GEMMA) method (Zhou and Stephens, 2012). GRAMMAR is a two-step method that first estimates the residuals from mixed model without a SNP effect and then treats these residuals as corrected phenotypes for GWAS by simple linear regression. GEMMA is an exact mixed model approach that tests for association efficiently by using the mixed model with a SNP effect at one step. The whole genomic relationship matrix used in RHM was also used to perform the GRAMMAR and GEMMA analyses. The minimum *P*-values of GWAS were recorded in each win100 replicate. The *P*-value of thresholds for genome-wide significance came from the Bonferroni correction accounting for 268,600 SNPs, and the power to achieve 5% genome-wide significance was calculated as the proportion of replicates with a significant association. The heritability at the most significant SNP was calculated assuming Hardy–Weinberg proportions for the SNP genotypes; SNP heritability at the SNP with the minimum *P*-value, *h*^2^_SNP_, was calculated as *h*^2^_SNP_ = 2*p*(1 − *p*)*b*^2^/σ^2^, where *p* was the SNP MAF, *b* was the SNP effect (regression coefficient estimated from the analysis), and σ^2^ was the residual variance for GRAMMAR and the phenotypic variance for GEMMA (Falconer and Mackay, 1996). An average value of SNP heritability across simulation replicates was calculated for the GRAMMAR and GEMMA analyses.

To investigate the power of RHM and other GWAS methods that consider several variants in a (gene) region simultaneously, we analyzed the data using three recently reported gene-based association tests. These GWAS methods implement gene-based association approaches which consider an association between a trait and all markers within a gene rather than each marker individually, and generate one new *P*-value as a representative value of the gene. These methods can account for the number of independent effects within a gene. Three gene-based association approaches were as follows:

A versatile gene-based test for genome-wide association studies (VEGAS): VEGAS proposed by Liu et al. (2010) sums the SNP-based chi-square test statistics from all the SNPs within a gene and then corrects the sum for LD to generate a gene-based test statistic. VEGAS requires the pairwise LD correlation matrix of the SNPs from HapMap genotype information calculated by the PLINK software (Purcell et al., 2007). In this study, a custom set of individual genotypes was used to estimate an LD correlation matrix by using genotype data from our population, instead of HapMap genotype information, because the selected region is not a gene locus. The VEGAS test was performed by using the *P*-values obtained from GEMMA analysis.

Sequence kernel association test (SKAT): As a kernel machine based test, SKAT proposed by Wu et al. (2011) aggregates genetic information across the region using a kernel function and uses a computationally efficient variance component test to test for association. This method has an advantage if the causal mutation is rare. SKAT's power is greater than that of several burden tests such as the cohort allelic sum test (Morgenthaler and Thilly, 2007). In this study, the GRAMMAR method was used obtain a phenotype adjusted for population stratification that was then used in SKAT analysis. We used the default beta (1, 25) weight in this study.

Canonical Correlation Analysis (CCA): Tang and Ferreira (2012) explored the gene-based association test using canonical correlation to test multiple SNPs for association with a single or multiple phenotypes measured in unrelated individuals. CCA removes any multicollinearity between SNPs by accounting for pairwise (LD) correlations and variance inflation factor, calculates canonical correlations between selected SNPs and phenotypes, and tests the significance of all canonical correlations. Tang and Ferreira (2012) showed that the power of this method was greater than that of GWiS (Huang et al., 2011) and single-SNP GWAS. We used the GRAMMAR-adjusted phenotypes as input in the CCA analysis.

In our simulated 300 “gene regions,” for the high_info group, RHM with win10, single-SNP GWAS by GEMMA and three gene-based association approaches were performed, and the power to achieve 5% genome-wide significance was calculated. For single-SNP GWAS, only GEMMA was performed in this analysis, because the power to detect QTL using GEMMA was greater than that obtained using GRAMMAR in all simulations (see Results), and the minimum *P*-value was calculated in a gene region. For the low_info group, there was no significant result for any methods in all simulations (see Results), and therefore results of these analyses are not presented.

### Regional heritability mapping

We performed RHM based on two-step variance component method described by Nagamine et al. (2012) using ASReml software (Gilmour et al., 2006). The mixed model is as follows;

(1) $$\text{y}=1_{\text{n}}\mu +\text{Xu}+\text{Zw}+\text{e}$$

where **y** is the vector of phenotypic values and **X** and **Z** are the design matrices for random effects. 1_*n*_ is a vector of 1s and μ is the mean. *u* ~ *N*(0, *G*σ^2^_*u*_) is the whole genomic additive genetic effect, *w* ~ *N*(0, *Q*σ^2^_*w*_) is the regional genomic additive genetic effect and *e* ~ *N*(0, *I*σ^2^_*e*_) is the residual effect. Matrices **G**, **Q**, and **I** are a whole genomic relationship matrix, a regional genomic relationship matrix using SNPs within the short region of genome, and an identity matrix, respectively. Elements of matrices **G** and **Q** are based on genomic kinship and inbreeding coefficient between individual *i* and *j* using identity by state (IBS), and element *f*_*ij*_ of both **G** and **Q** is defined as follows,

$$\begin{array}{l} \;f_{ij}=\frac{2}{n}\text{​}\sum_{k=1}^{n}\frac{\left(x_{ik}-p_{k})\text{​}(x_{jk}-p_{k}\right)}{p_{k}(1-p_{k})},\;(i\neq j)\\ f_{ij}=1+\frac{1}{n}\text{​}\sum_{k=1}^{n}\frac{Obs{\left(\#hom\right)}_{ik}-E{\left(\#hom\right)}_{k}}{1-E{\left(\#hom\right)}_{k}},\;(i=j) \end{array}$$

where *x*_*ik*_ (*x*_*jk*_) is the genotype of the *i*-th (*j*-th) person at the *k*-th SNP (coded as 0, 0.5, and 1 for AA, AB, and BB, respectively). Here *n* represents the total genomic SNPs for matrix **G** or the number of SNPs in the region for matrix **Q**. The frequency *p*_*k*_ is for the *B* allele at the *k*-th SNP, and *n* is the number of SNPs. *Obs(#hom)_ik_* and *E(#hom)_k_* are the observed and expected number of homozygous genotypes in the *i*-th person at the *k*-th SNP. Regional heritability *h*^2^_*RH*_ and genome heritability *h*^2^_*GH*_ are calculated as follows,

$$\begin{array}{l} h_{RH}^{2}=\frac{\sigma_{w}^{2}}{\sigma_{u}^{2}+\sigma_{w}^{2}+\sigma_{e}^{2}}\\ h_{GH}^{2}=\frac{\sigma_{u}^{2}}{\sigma_{u}^{2}+\sigma_{w}^{2}+\sigma_{e}^{2}} \end{array}$$

where σ^2^_*u*_, σ^2^_*w*_, σ^2^_*e*_ are whole genome additive genetic variance, regional genomic additive variance, and residual variance, respectively.

### Test statistics and their distribution

To test for the presence of QTL effect against the null hypothesis (no regional variance) at a test region (window), the likelihood ratio test statistics (LRT) = −2 ln(*L*_0_–*L*_1_) was calculated, where *L*_0_ and *L*_1_ represent the likelihood values under the hypothesis of no presence (*H*_0_) and presence (*H*_1_) of regional variance, respectively. The *L*_1_ was calculated by using the model (1), and the *L*_0_ was calculated by using the following mixed model (2) that does not include regional genomic additive genetic effect from the model (1).

(2) $$\text{y}=1_{n}\mu +\text{Xu}+\text{e}$$

Statistical theory states that the LRT follows a χ^2^ distribution with the degrees of freedom equal to the number of random parameters being tested (Wilks, 1938). However, for testing a single variance component in a REML context, the asymptotic distribution of the LRT under the null hypothesis follows a mixture of χ^2^ distributions with different degrees of freedom (e.g., Visscher, 2006). Hence for the RHM method, the LRT follows a 50:50 mixture distribution, where one mixture component is a peak at 0 and the other component is a χ^2^_1_ distribution (Nagamine et al., 2012). In this study, phenotypes under the null hypothesis were generated, and LRTs for each non-overlapping win100 were calculated to obtain an empirical distribution of −log_10_(*P*-value) under the null hypothesis and compared with the theoretical distribution. The results show that the 50:50 mixture distribution is more appropriate (see Figure S2 in Supplementary Material).

### Analyses of real population data on three biometrical eye traits

To illustrate the applicability of RHM in the real population data, we considered three eye traits measured in four populations [three Croatian (CROATIA-Vis, CROATIA-Korcula, CROATIA-Split) and one from Orkney (ORCADES), including axial length (AL), central corneal thickness (CCT), and spherical equivalent refraction (SER)]. These are quantitative endophenotypes related to common eye disorders; AL and SER are related to incidence of myopia and hyperopia and CCT is related to the incidence of corneal disorders and probably glaucoma. All cohorts have contributed to large single-SNP GWAS meta-analyses efforts studying these phenotypes (Lu et al., 2013; Verhoeven et al., 2013). All the Croatian cohorts (that will be referred from here as Vis, Korcula, and Split) received ethical approval from the Ethics Committee of the Medical School, University of Split and the NHS Lothian (South East Scotland Research Ethics Committee). The ORCADES cohort, referred to as Orkney from now on received ethical approval from the NHS Orkney Research Ethics Committee and North of Scotland Research Ethics Committee. All studies followed the tenets of the Declaration of Helsinki and all participants gave written informed consent. A total of 2245 individuals for AL, 2261 individuals for CCT, and 2251 individuals for SER were measured, and descriptive statistics for these three traits were shown in Table S1 in Supplementary Material. The Vis cohort genotyping was performed using the Illumina HAP300v1 SNP array, the Korcula and Split cohorts were genotyped using the Illumina HAP370CNV SNP array, and the Orkney cohort used the Illumina HumanHap300 beadchip. A total of 3210 individuals in four populations were genotyped (the number of individuals in each population is shown in Table S1 in Supplementary Material). A total of 344,065 SNPs with overlap among four populations were assessed by the same protocol as above, and 272,315 SNPs on autosomal chromosomes passed the quality control (the number of SNPs in each chromosome is shown in Table S2 in Supplementary Material). We performed single-SNP GEMMA analysis and RHM across the whole genome to detect any significant regions. To account for non-genetic effects in these two analyses, population, and sex were included as fixed effects, and age (and height in AL) was used as a covariate in these analyses. The significance threshold value for single-SNP GEMMA was determined by Bonferroni correction with 272,315 SNPs. For RHM, we applied a two-step approach to reduce computation. At first, RHM with win100 was performed across all autosomes. The window was shifted every 50 SNPs to overlap a region, and a total of 5412 windows were tested across chromosomes. In the second step, the top 100 win100s with higher LRT were selected from all 5412 windows, and then each win100 was divided equally into 10 win10s and 5 win20s, and RHM with win10 and win20 was performed. To evaluate the power of other GWAS methods, the windows with *P*-value < 1.0 × 10^−5^ in RHM analyses were then analyzed by three gene-based association approaches (VEGAS, CCA, and SKAT), and the window was assumed as a “gene region” in these methods. The methodologies of these gene-based association approaches were the same as above. To determine the significance threshold value of RHM and the three gene-based association approaches with win20 and win10, the Bonferroni correction was applied by using 27,060 and 54,120 windows, respectively.

## Results

### Imputed SNPs

**Figure 1:**
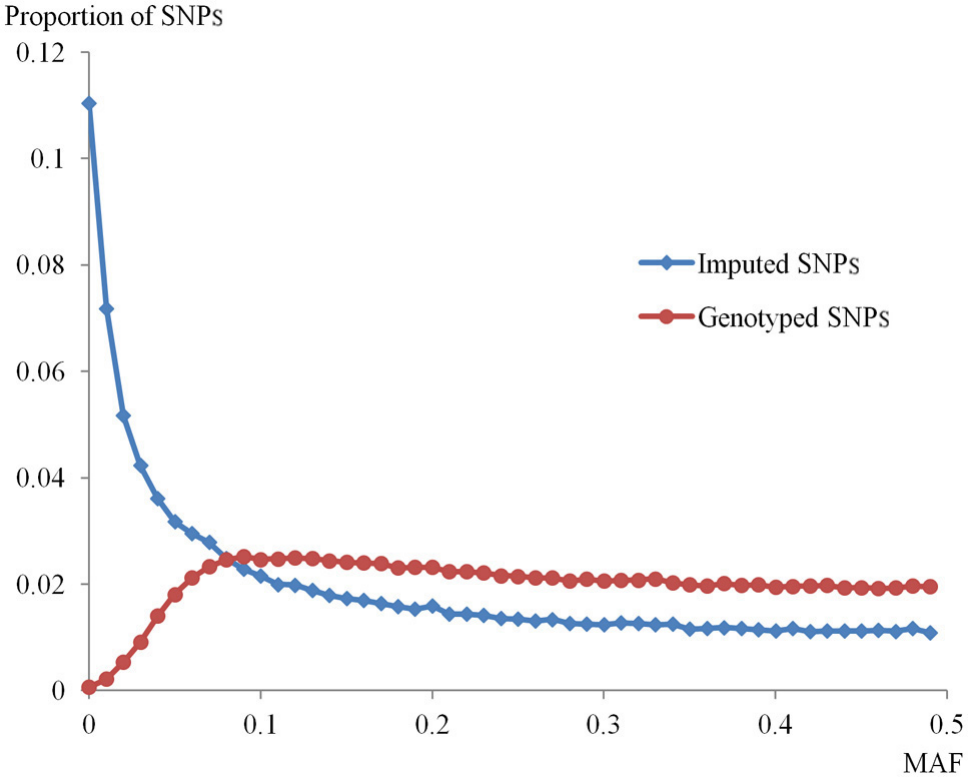
**Distribution of minor allele frequencies (MAFs) for genotyped and imputed SNPs**. The distributions of MAF for imputed SNPs within the high_info group and for genotyped SNPs in this population are shown. The x-axis indicates the MAF of both groups of SNPs. The y-axis represents the proportion of SNPs in each MAF category.

After removing markers with the exclusion criteria we have described, a total of 6,704,137 SNPs in the high_info group and 3,793,540 SNPs in the low_info group were available. Table 2 shows the summary of imputed SNP number within a win10 region for low_info and high_info groups. In the low_info group, almost all SNPs had low MAF, and therefore only SNPs with low MAF were used in the simulation. In the high_info group, 45% of SNPs had low MAF and 55% of SNPs had high MAF. The density distributions of MAF for imputed SNPs within the high_info group and for genotyped SNPs are plotted in Figure 1. The MAF distribution shows a very low ratio of genotyped to imputed SNPs at low MAF, pointing to the difficulty of capturing genetic variance if imputed SNPs at low MAF are assumed to be QTL.

**Table 2:** **Total number of imputed SNPs and summary of SNP number in a window containing 10 genotyped SNPs (win10) for two different IMPUTE2-info scores in the simulation study**.

**IMPUTE2-info score**	**Total number of SNPs**		**Number of SNPs in win10**		
			**Total**	**Low MAF**	**High MAF**
Low_info group	3,793,540	Mean	141	140	1
		Max	3749	3241	508
		Min	0	0	0
High_info group	6,704,137	Mean	250	112	138
		Max	5126	1965	3161
		Min	0	0	0

### The power of RHM and single-SNP GWAS in the 100-SNP window

**Figure 2:**
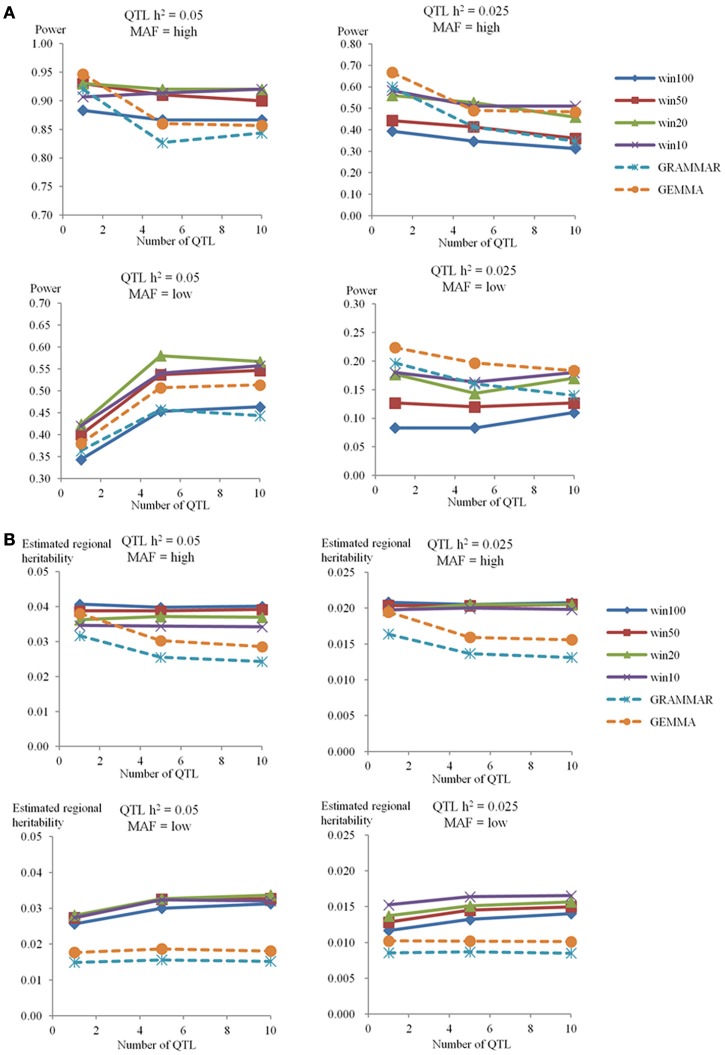
**The power to achieve 5% genome-wide significance and estimated regional heritability in the 100-SNP window**. The powers **(A)** and estimated regional heritabilities **(B)** in the simulation study for genome heritability 0.4 were calculated by RHM with four different window sizes (100 SNPs as win100, 50 SNPs as win50, 20 SNPs as win20, and 10 SNPs as win10), and two single-SNP GWAS methods (GRAMMAR and GEMMA) in the different situations. The number of QTL is on the x-axis, and the power to detect QTL **(A)** or the estimated regional heritability **(B)** are on the y-axis. Each graph shows the different situations for genome heritability 0.4 (QTL heritability is 0.05 or 0.025, and MAF is high or low).

In the low_info group, there was no significant replicate in all simulations, indicating that the power was low for both methods. For the high_info group, the power to detect QTL for the phenotype with genome heritability 0.4 is shown in Figure 2. For RHM, as the number of QTL increased, the power to detect QTL was almost constant in all simulated scenarios, except when the QTL had low MAF and 0.05 QTL heritability. For RHM, using smaller window sizes (win10 and win20) yielded greater power than using larger window sizes (win50 and win100), and the difference in power among window sizes was almost the same for different numbers of simulated QTL. There was no significant difference in power among simulations with different genome heritability (see Figure S3 in Supplementary Material). For single-SNP GWAS, as the number of QTL increased, the power to detect QTL decreased, except for QTL with low MAF and 0.05 QTL heritability, where it increased as was also the case for RHM. Changes in genome heritability, had no large impact in power for the GEMMA analyses, but the power of GRAMMAR analyses decreased as the genome heritability increased (see Figure S3 in Supplementary Material). The difference in power between RHM and single-SNP GWAS varied with QTL MAF. For high MAF QTL, the power of single-SNP GWAS was greater than that of RHM when the number of QTL was one. But as the number of QTL increased, the power of RHM was greater than that of single-SNP GWAS. For low MAF, the power of RHM was higher than that of single-SNP GWAS for 0.05 QTL heritability, but lower for 0.025 QTL heritability for all numbers of QTL.

### The estimated regional heritability of RHM and single-SNP GWAS in the 100-SNP window

In the high_info group for a genome heritability of 0.40, the estimated regional and genome heritabilities are shown in Figure 2 and Table S3 in Supplementary Material, respectively. For all methods the mean heritability captured was less than that actually simulated but RHM generally captured a substantially greater proportion than GEMMA, the best of the single SNP methods, although RHM and GEMMA captured a similar proportion of simulated heritability when there was a single high MAF QTL. For RHM, as the number of QTL increased, the estimated regional heritability remained almost constant (averaging about 80% of the amount simulated) for QTL with high MAF, but it increased slightly with the number of QTL at low MAF (averaging around 60% of the amount simulated). Overall, there were no large differences in the amount of heritability captured by RHM using different window sizes and no overall trend in the size of window capturing most heritability. There was also no big difference for estimated regional heritability with different genome heritabilities (see Figure S4 in Supplementary Material). For single-SNP GWAS, as the number of QTL increased, the estimated regional heritability decreased for high MAF QTL, but was almost constant for low MAF QTL. On average GEMMA estimates of the QTL heritability were almost 80% of that simulated for a single high MAF QTL, but the estimates dropped to around 60% of the simulated values for 5 or 10 high MAF QTL and were only about 40% of the simulated values for 1, 5, and 10 simulated low MAF QTL. Varying the genome heritability produced no big difference in the QTL heritability captured by GEMMA. As the simulated genome heritability increased, the regional heritability estimated by GRAMMAR decreased (see Figure S4 in Supplementary Material). Table S3 in Supplementary Material also showed the genome heritability estimated by model (2). The genome heritability estimated for high MAF was close to the simulated value, but genome heritability was underestimated for low MAF QTL.

### The power of RHM and other methods in the gene region

**Figure 3:**
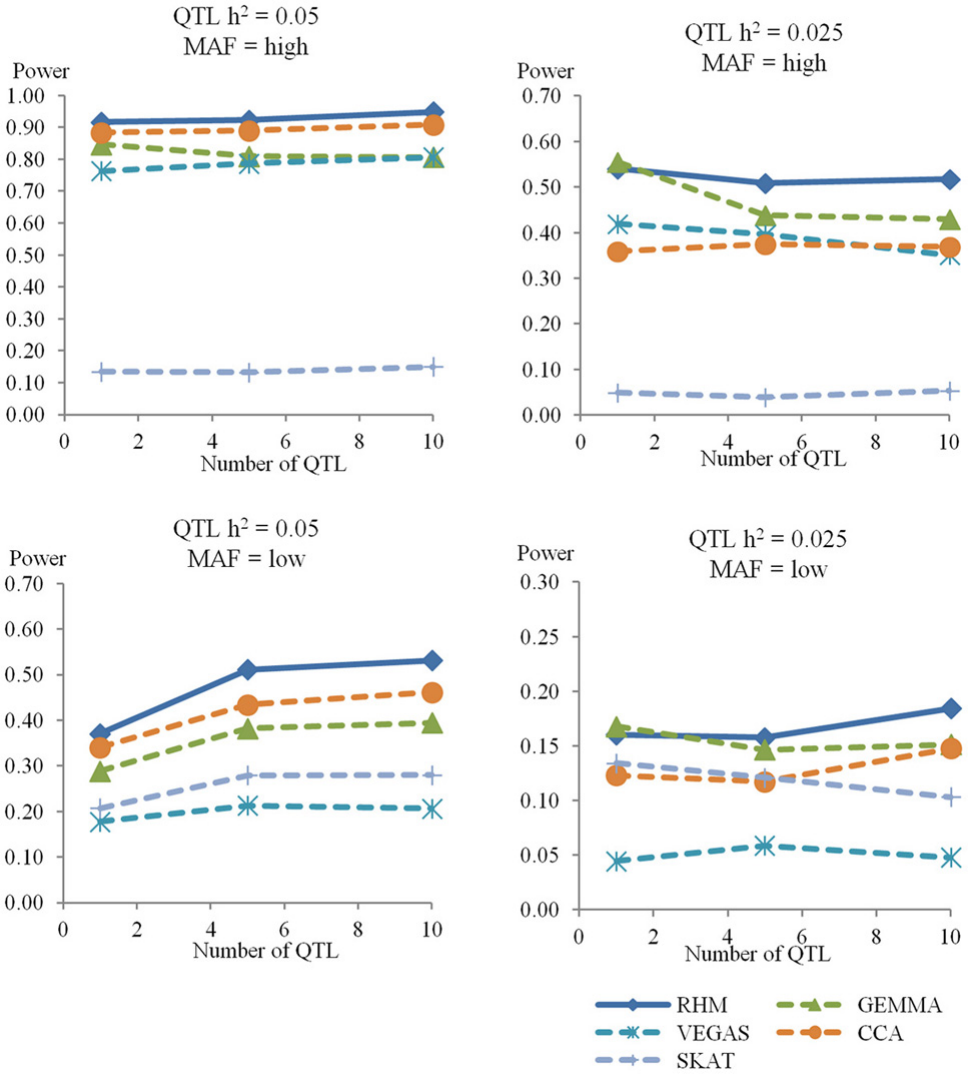
**The power to achieve 5% genome-wide significance in the gene region**. The powers in the simulation study for genome heritability 0.4 were calculated by RHM with window size 10 (RHM), single-SNP GWAS (GEMMA), and three gene-based association approaches (VEGAS, CCA, and SKAT) in the different situations. The number of QTL is on the x-axis, and the power to detect QTL is on the y-axis. Each graph shows the different situations for genome heritability 0.4 (QTL heritability is 0.05 or 0.025, and MAF is high or low).

For the genome heritability of 0.40, Figure 3 shows the results of power for RHM with win10, single-SNP GWAS (GEMMA), and three gene-based association approaches (VEGAS, SKAT, and CCA) in a gene region. The power of RHM was higher than that of all other methods for most simulation conditions, with the exception of the single QTL with 0.025 heritability, for which GEMMA had slightly higher power than RHM. As the number of QTL increased, the power to detect QTL generally remained almost constant or slightly reduced in all methods, but it increased slightly for all methods with low MAF and 0.05 QTL heritability. As for the other methods, CCA was the most powerful for QTL with high heritability, and GEMMA was the most powerful for QTL with low heritability. The power of VEGAS and SKAT was the lowest for QTL with low MAF and high MAF, respectively. The magnitude of the genome heritability had no great impact of on the power of RHM or GEMMA based methods (single-SNP GWAS and VEGAS), but the power of methods using GRAMMAR-adjusted phenotype (SKAT and CCA) decreased as the genome heritability increased (see Figure S5 in Supplementary Material).

**Figure 4:**
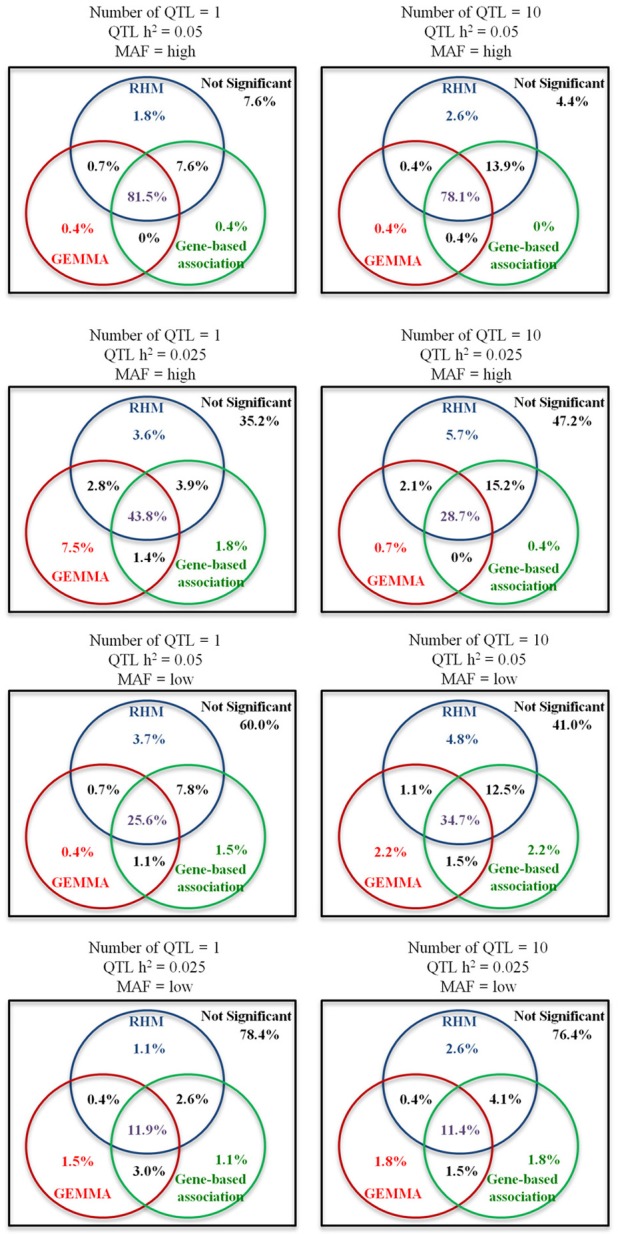
**Venn diagrams for comparisons of the significantly associated gene regions identified by three different methods**. The percentages in circles are the proportions of the significantly associated gene regions identified by three different methods: blue circles for RHM, read circles for single-SNP GWAS (GEMMA), and green circles for gene-based association approaches including VEGAS, CCA, and SKAT (Gene-based association). Percentages in purple represent the significant replicates shared by all three methods, percentages in black represent the significant replicates shared only by two methods, and percentages in other colors are the significant replicates identified only by the corresponding method. The percentages in the squares are the proportion of not-significantly associated replicates. Each Venn diagram shows the different situations for genome heritability 0.4 (Number of QTL is 1 or 10, QTL heritability is 0.05 or 0.025, and MAF is high or low).

The Venn diagrams for comparisons of the significantly associated regions identified by three different methods (RHM, GEMMA, and gene-based association approach) are shown in Figure 4. As the number of QTL increased, the probability that QTL were detected only by RHM increased. For GEMMA and gene-based association approaches, as the number of QTL increased, the power to detect QTL by each method increased for low MAF but decreased or stayed constant for high MAF. In addition, RHM identifies some additional loci, even where GEMMA has higher power than RHM as is the case for the single QTL with 0.025 heritability. By using RHM and GEMMA, more than 90% of the QTL which were detected in all methods can be captured in all simulations.

### Analyses of real population data on three biometrical eye traits

**Figure 5:**
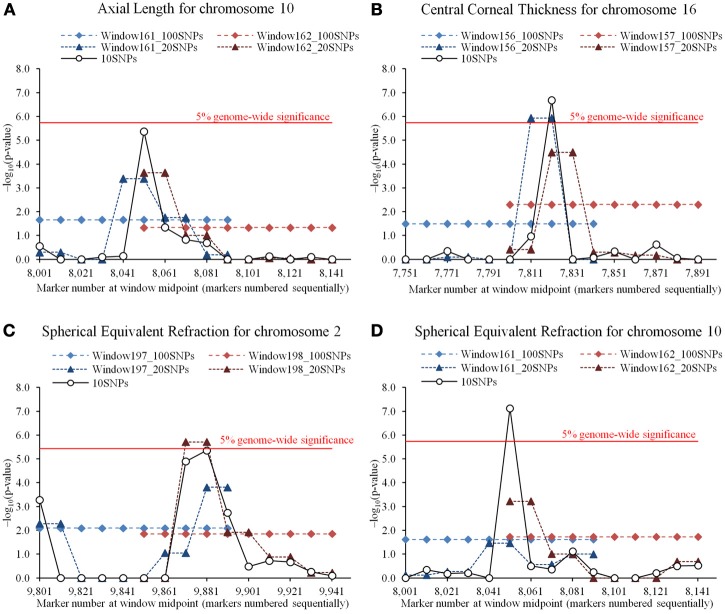
**Comparisons of regional heritability mapping (RHM) among different window sizes on a significant region for three eye traits**. Comparisons shown on the results of higher −log_10_(*P*-value) (>5.0) for axial length (AL), central corneal thickness (CCT), and spherical equivalent refraction (SER). **(A)** The results of AL on chromosome 10. The plot shows the −log_10_(*P*-value) of 100-SNP-window number 161 for RHM with win100 (Window161_100SNPs) and win20 (Window161_20 SNPs), 100-SNP-window number 162 for RHM with win100 (Window162_100SNPs) and win20 (Window162_20SNPs), and 100-SNP-window numbers 161 and 162 for RHM with win10 (10SNPs). The red horizontal line is drawn at the 5% genome-wide significance for RHM with win10. **(B)** The result of CCT on chromosome 16. The plot shows the −log_10_(*P*-value) of 100-SNP-window number 156 for RHM with win100 (Window156_100SNPs) and win20 (Window156_20 SNPs), 100-SNP-window number 157 for RHM with win100 (Window157_100SNPs) and win20 (Window157_20SNPs), and 100-SNP-window numbers 156 and 157 for RHM with win10 (10SNPs). The red horizontal line is drawn at the 5% genome-wide significance for RHM with win10. **(C)** The result of SER on chromosome 2. The plot shows the −log_10_(*P*-value) of 100-SNP-window number 197 for RHM with win100 (Window197_100SNPs) and win20 (Window197_20 SNPs), 100-SNP-window number 198 for RHM with win100 (Window198_100SNPs) and win20 (Window198_20SNPs), and 100-SNP-window numbers 197 and 198 for RHM with win10 (10SNPs). The red horizontal line is drawn at the 5% genome-wide significance for RHM with win20. **(D)** The result of SER on chromosome 10. The plot shows the −log_10_(*P*-value) of 100-SNP-window number 161 for RHM with win100 (Window161_100SNPs) and win20 (Window161_20 SNPs), 100-SNP-window number 162 for RHM with win100 (Window162_ 100SNPs) and win20 (Window162_20SNPs), and 100-SNP-window numbers 161 and 162 for RHM with win10 (10SNPs). The red horizontal line is drawn at the 5% genome-wide significance for RHM with win10.

Quantile-quantile plots for the GEMMA results shown in Figure S6 in Supplementary Material demonstrate that population stratification was successfully accounted by this method. Genome-wide plots of *P*-values for AL, CCT, and SER by GEMMA are shown in Figure S7. For GEMMA, two significant SNPs were detected for CCT, the significant SNPs being rs1536482 (*P*-value = 1.0 × 10^−7^) on chromosome 9 and rs12447690 (*P*-value = 3.3 × 10^−11^) on chromosome 16. These hits represent the *RXRA-COL5A1* and *ZNF469* loci as reported by Vitart et al. (2010) and both replicated in multiple studies (Lu et al., 2013). For RHM, the top 100 win100s with higher LRT were selected for further analysis using win10 and win20. The results from these latter analyses that gave *P*-values < 1.0 × 10^−5^ are given in Figure 5 and Table 3. For AL, there was no significant region, but a novel region with a *P*-value < 1.0 × 10^−5^ was detected on chromosome 10 by RHM with win10. For CCT, there was a significant region on chromosome 16 that included the significant SNP detected by GEMMA and with the *ZNF469* gene located near this region (Figure S8 in Supplementary Material). For SER, there were two significant novel regions [unreported in the largest single-SNP GWAS meta-analyses published by Verhoeven et al. (2013); Kiefer et al. (2013)] detected by RHM with win20 on chromosome 2 and with win10 on chromosome 10, this latter was the same region as detected for AL, a trait phenotypically correlated to SER. On chromosome 2, the two genes (*CREG2* and *RNF149* loci) and four genes (CREG2, *RNF149*, *SNORD89*, and *C2orf29* loci) were located within win10 with the lowest *P*-value and the significant win20, respectively, and there was no coding gene in the significant region of chromosome 10 (Figure S8 in Supplementary Material). On chromosome 9, the *RXRA-COL5A1* locus detected by GEMMA was not significant by RHM. To evaluate the power of three gene-based association approaches, these windows were analyzed, and the results were shown in Table 3. The significant region was detected by VEGAS on chromosome 16, but there were no other significant regions detected by VEGAS or other methods (SKAT and CCA).

**Table 3:** **Summary of the significant regions for Regional heritability mapping (RHM) and other methods in Axial Length, Central Corneal Thickness, and Spherical Equivalent Refraction**.

**Trait^1^**	**Chromosome**	**Window size**								**RHM**				**Other methods**			
			**SNP number**		**SNP name and position**		**MAF**					**Heritability**		***P*-value^4^**			
			**Start**	**End**	**Start**	**End**	**Min**	**Mean**	**Max**	**LRT^2^**	***P*-value^3^**	**Regional**	**Genome**	**GEMMA^5^**	**VEGAS**	**CCA**	**SKAT**
AL	10	10	8051	8060	rs1877591	rs11002552	0.08	0.14	0.28	21.2	4.2 × 10^−6^	0.049	0.438	4.7 × 10^−3^	6.9 × 10^−3^	1.4×10^−2^	6.2×10^−2^
					79,784,171	79,870,936											
CCT	9	10	13,711	13,720	rs1536482	rs4304399	0.05	0.22	0.49	8.1	4.4 × 10^−3^	0.013	0.818	1.0 × 10^−7^^**^	1.1 × 10^−3^	1.8 × 10^−4^	9.8 × 10^−1^
					134,666,473	134,746,881											
	16	20	7811	7830	rs7403882	rs8044502	0.08	0.27	0.52	23.6	1.2 × 10^−6^^**^	0.026	0.799	3.3 × 10^−11^^**^	1.0 × 10^−6^^**^	4.2 × 10^−5^	1.8 × 10^−1^
					86,639,452	87,003,009											
	16	10	7821	7830	rs12597413	rs8044502	0.16	0.34	0.52	27.0	2.1 × 10^−7^^**^	0.026	0.795	3.3 × 10^−11^^**^	0^**^	3.0 × 10^−6^	7.5 × 10^−1^
					86,782,559	87,003,009											
SER	2	20	9871	9890	rs4851411	rs3923053	0.10	0.25	0.53	22.6	2.0 × 10^−6^^**^	0.150	0.349	6.9 × 10^−2^	6.0 × 10^−1^	9.2 × 10^−1^	7.0 × 10^−1^
					101,260,596	101,444,879											
	2	10	9881	9890	rs7568067	rs3923053	0.10	0.21	0.48	21.1	4.4 × 10^−6^	0.112	0.369	6.9 × 10^−2^	5.1 × 10^−1^	5.5 × 10^−1^	8.1 × 10^−1^
					101,385,329	101,444,879											
	10	10	8051	8060	rs1877591	rs11002552	0.08	0.14	0.28	28.9	7.5 × 10^−8^^**^	0.092	0.362	2.9 × 10^−2^	7.2 × 10^−3^	2.0 × 10^−1^	1.4 × 10^−1^
					79,784,171	79,870,936											

1 *AL, Axial Length; CCT, Central Corneal Thickness; SER, Spherical Equivalent Refraction*.

2 *Likelihood ratio test statistics*.

3 *For guidance, the 5% genome-wide significance for RHM with window size 10 (P-value = 1.8 × 10^−6^) and window size 20 (P-value = 3.7 × 10^−6^)*.

4 *For guidance, the 5% genome-wide significance for GEMMA (P-value = 1.8 × 10^−7^), 5% genome-wide significance for gene-based association approaches with window size 10 (P-value = 9.2 × 10^−7^) and window size 20 (P-value = 1.8 × 10^−6^)*.

5 *The minimum P-value in the window was selected*.

6** *5% genome-wide significance level*.

## Discussion

Nagamine et al. (2012) introduced a new variance component-based mapping methodology, referred to as regional genomic relationship mapping or RHM, to localize some of the genetic variation that cannot be detected by single-SNP GWAS analyses. Here, we study in depth the implementation and power of RHM in a range of circumstances. In particular, we describe the power to detect regions harboring different numbers of QTL with different MAFs (common and rare) explaining different proportions of the trait variance and the accuracy for estimating regional heritability. We also compare these results to those obtained using a range of single-SNP GWAS and gene-based association approaches. In addition, we applied RHM to the analysis of eye traits to show the effectiveness of this method.

Our simulation was based on real genotype data from a human population in an attempt to accurately account for LD found in real populations between marker SNP and QTL. Using imputed SNPs as the simulated QTL allowed us to generate a number of QTL in a region at both high and low MAF whilst retaining the genotyped SNPs as the markers for analysis. As might be expected, in our analyses of QTL based on poorly imputed SNPs (information score <0.5) no method was able to detect the simulated QTL. With QTL simulated based on well-imputed SNPs (information score >0.7) all methods we used had some power and often they were quite similar. Nonetheless, overall RHM was similar or greater in power to detect QTL than single SNP GWAS and had greater power than other gene-based methods. In particular, RHM had greater power to detect low MAF QTL and/or multiple independent QTL effects acting in a region than any of the methods of single-SNP GWAS and gene-based association approaches we tested, especially when RHM was performed using smaller analysis window sizes. RHM also captured a larger proportion of the QTL variance caused by multiple independent QTL and/or low MAF QTL. Importantly, for QTL with low MAF, RHM was capable of capturing more of the QTL variance than single-SNP GWAS for all magnitudes of QTL heritability.

GEMMA had slightly higher power than RHM when we simulated a single QTL with 0.025 QTL heritability. However, even in this case RHM found additional loci not detected by GEMMA. RHM also had greater power to detect QTL than GEMMA when several QTL in a region contribute trait variation and all have low MAF.

The effect of QTL MAF was evaluated by simulating QTL in the low MAF (MAF < 0.1) and high MAF (MAF ≥ 0.10) groups. As the number of QTL per window increased, the power to detect QTL also increased when the QTL had low MAF and 0.05 QTL heritability (Figure 2 and Supplementary Figure S3). When a single low MAF QTL is randomly selected, it is likely to be very rare (as very rare SNPs are more common within the low MAF group than moderately rare ones, see Figure 1) and hence not well-captured by genotyped SNPs. When multiple (5 or 10) low MAF QTL are selected, one or more of the less rare ones within the low MAF group may well be chosen. These will contribute much of the variance and are likely to be better captured by genotyped SNPs leading to increased power when there were more QTL per window.

The power to detect QTL by RHM was greater than that of the three gene-based association approaches studied. We found that these gene-based association methods are strongly affected by the QTL MAF. The power of VEGAS and SKAT was greatly decreased for low MAF or high MAF QTL, respectively. SKAT was developed as a rare-variant association test (Wu et al., 2011), and uses a weighting scheme that upweights the contribution of rare variants and downweights the contribution of common variants in its default setting. Therefore, this default setting would be less powerful when variants have high MAF. VEGAS corrects the test statistics by LD between genotyped SNPs (Liu et al., 2010), and this correction might lose the power in the condition with low MAF because of incomplete LD between genotyped SNPs and QTL. In addition, these methods are also affected by the genome heritability. In this simulation, GRAMMAR-adjusted phenotypes are used to correct the effect of population stratification in SKAT and CCA, because these methods are not designed within a mixed model framework and cannot readily account for family relatedness among samples. The power for high genome heritability is lower than that for low genome heritability in these methods. But RHM was also more powerful than all gene-based association approaches at low genome heritability. For comparisons of the significant regions identified by RHM, GEMMA, and gene-based association approaches, more than 90% of the QTL can be captured by only RHM and GEMMA. Therefore, we suggest that RHM should be used as the complementary method which detects a different set of QTL when the power to detect QTL is not complete.

RHM has the potential to capture some of the “missing heritability.” Yang et al. (2010) estimated that common SNP variation explained more than half of the expected heritability of human height, and suggested that missing heritability is due to imperfect LD between genotyped SNPs and causal variants. Yang et al. (2010) also simulated a quantitative trait by randomly sampling causal variants from the SNPs with MAF ≤ 0.10, and showed that estimated genome heritability was underestimated in comparison with the true genome heritability. In this study, the genome heritability estimated by model (2) for low MAF QTL was also underestimated in comparison with that for high MAF QTL. However, RHM captured more QTL variance with low MAF QTL than single-SNP GWAS and hence may capture heritability missed by single SNP GWAS.

Many explanations for the missing heritability have been suggested: a large number of common variants with small effect, a moderate number of rare variants with large effect, and some of combination of genotypic, environmental and epigenetic interactions (Manolio et al., 2009; Gibson, 2012). In this study, we show that RHM has the potential to explain some of the missing heritability through identification of trait-associated low MAF QTL by using common SNPs. However, some genetic variance could not be captured as some of the QTL variance is not in LD with individual common SNPs. An alternative method to capture QTL variance using common SNPs would be haplotype-based association, and some of the unknown low MAF QTL might be recovered by re-constructing haplotypes using common SNPs. However, some rare variants will be unique to particular populations and it will be difficult to detect QTL which are in linkage equilibrium with common SNPs. In this case we suggest that using exome sequencing or exome genotyping arrays combined with RHM on these types of data has the potential to capture even more of the missing variance.

In the study of real population data, some significant regions were detected by single-SNP GWAS, RHM or gene-based association approaches, corresponding to known loci but, additionally, two loci were newly identified, only by RHM, for SER. For the first one on chromosome 2, the *P*-value of win20 (SNP number 9871–9890) was lower than that of win10 (SNP number 9881–9890), and the win20 had high regional heritability 0.150 and contains four loci genes not previously implicated in refractive error control. There, multiple independent QTL of low MAF might be located on this narrow segment region. For the putative second novel SER locus, on chromosome 10, the regional signal was also suggestive for the phenotypically correlated trait AL making it unlikely to be a false positive finding. The closest genome-wide significant hit reported in the large GWAS meta-analyses of similar traits (SER or myopia) is a megabase away [Myopia GWAS SNP rs6480859 reported by Kiefer et al. (2013)] and although it is unlikely that the two findings reflect the same causal signal, they may highlight the same gene. Further analyses using other populations will be needed to validate these findings but this may be difficult if the variants are rare and their contribution to the trait variance large enough to be detectable in specific populations only. Functional analysis of the regions highlighted may also help confirming involvement of these regions. In this study, the significant *RXRA-COL5A1* CCT lead SNP on chromosome 9 detected by single-SNP GWAS was not detected by RHM. These mirrored the same trend as our simulation study, and also suggest that RHM should be an important complementary method to single-SNP GWAS, where multiple variants of low effect size and a range of MAFs may be segregating.

Nagamine et al. (2012) introduced RHM approach, and we present the effectiveness and implementation of RHM by assuming QTL in a narrow segment region, evaluating the impact of window size, and comparing with other single-SNP GWAS and gene-based association approaches under many different conditions. In addition, we detected some additional loci which were not detected by single-SNP GWAS and gene-based association approaches in real population data. We suggest in this study that RHM using common SNPs has the potential to explain some of missing heritability by capturing QTL variance with low MAF and localizing multiple independent QTL in a segment region. In conclusion, the results reported in this study support that RHM is more powerful to detect QTL and capture QTL variance than other single-SNP GWAS and gene-based association approaches under most conditions in populations structured similarly to those we studied, which include both related and unrelated individuals.

## Author note

For software capable of implementing Regional Heritability Mapping (RHM) analyses in populations of related and/or unrelated individuals, see REACTA: Regional Heritability Advanced Complex Trait Analysis at http://www.epcc.ed.ac.uk/projects-portfolio/reacta.

### Conflict of interest statement

The authors declare that the research was conducted in the absence of any commercial or financial relationships that could be construed as a potential conflict of interest.

## Supplementary material

Figure S1 **Average *r*^2^-value plotted against inter-marker distance and Correlation plot**. To evaluate the quality of SNP imputation in this study, the difference of linkage disequilibrium (LD) between exome SNP and imputed SNP was investigated to evaluate whether the relationship between imputed SNPs is based on actual LD in this population or linkage equilibrium (LE). A total of 820 DNA samples from 898 Korcula samples were genotyped using the Illumina HumanExome-12v1 SNP array, that genotypes in excess of 250,000 exonic variants. These exome SNPs were then assessed by the exclusion criteria of minor allele frequency (MAF) <0.0005, call rate <0.98 and Hardy–Weinberg Equilibrium (HWE) < 1.0 × 10^−6^; SNPs included in Illumina CNV370 array were also excluded. A total of 7283 SNPs which were included in the low_info group and 25,313 SNPs in the high_info group were extracted from the exome array data. We estimated *r*^2^, a measure of LD, for all segregating pairs of SNPs less than 10 Mbp apart in each of these groups using the PLINK software (Purcell et al., 2007). Average *r*^2^-values for a given inter-marker distance, with markers distances grouped in 250 bp bins, were calculated in each autosome and plotted for each group. For the high_info group, *r*^2^-values for low MAF (MAF <0.10) and high MAF (MAF ≥ 0.10) SNPs were also calculated and plotted. The imputed SNPs for individuals with corresponding exome SNP data were extracted from the imputed SNP data, and *r*^2^-values were calculated as above. The correlation of *r*^2^-values obtained from genotyped SNPs (i.e., exome array data) and imputed SNPs was also estimated. The average *r*^2^-value was plotted against inter-SNP distance for exome SNPs and imputed SNPs in **(A)**, and the correlation of *r*^2^-value between exome SNPs and imputed SNPs was also plotted in **(B)**. In the high_info group, 12,636 SNPs with low MAF and 12,677 SNPs with high MAF were also used to calculate *r*^2^-values separately and then plotted. In the low_info group, there was no relationship between *r*^2^-value and marker distance, and no correlation of *r*^2^-value between exome SNPs and imputed SNPs. This result indicates that a high proportion of these exome and imputed SNPs in low_info group are estimated to be in linkage equilibrium (LE). On the other hand, in the high_info group, the *r*^2^-value in shorter inter-marker distances was higher than that in greater inter-marker distances, and there was high correlation of *r*^2^-values between exome SNPs and imputed SNPs. For the high_info group, the results within each MAF showed the same trend as the result for all SNPs and are not shown. The distribution and correlation of *r*^2^-values for high MAF was tighter than that for low MAF. In addition, the slopes of correlation in the high_info group were about 1.0 in all results, and the magnitude of LD between exome SNP and imputed SNPs was almost the same. This result indicates that the LD structure of the real population is still preserved in these imputed SNPs. **(A)** Average *r*^2^-value plotted against inter-marker distance for exome SNPs and imputed SNPs. The inter-marker distance grouped in 250 bp bins is on the x-axis, and average *r*^2^-value is on the y-axis. Each figure shows the results of SNPs with low MAF in the low_info group, and all SNPs, SNPs with low MAF, and SNPs with high MAF the in high_info group. **(B)** Correlation plot between average *r*^2^-values of exome SNPs and imputed SNPs. The average *r*^2^-value of imputed SNPs is on the x-axis, and the average *r*^2^-value of exome SNPs is on the y-axis. Each figure shows the results of SNPs with low MAF in the low_info group, and all SNPs, SNPs with low MAF, and SNPs with high MAF in the high_info group.

Figure S2 **Quantile-quantile plot of the *P*-values for Regional Heritability Mapping (RHM) with 100-SNP-windows**. For each value of simulated genome heritability (0.20, 0.40, and 0.8), the phenotype was generated, and then genome heritability was estimated by using model (2). The estimated genome heritabilities were 0.20 ± 0.07, 0.40 ± 0.07, and 0.80 ± 0.06 for the simulated values of 0.20, 0.40, and 0.80, respectively. We then performed RHM analyses using 100-SNP window (win100) using these phenotypes, to obtain empirically a distribution of test statistics under the null hypothesis. For each genome heritability, a quantile-quantile plot of the *P*-values of the RHM analyses with win100 assuming that they follow either a 50:50 mixture distribution of a χ^2^_1_ and a pick at 0 or a χ^2^_1_ distribution are shown. Results of analysis of generated phenotypes with genome heritability = 0.2, 0.4, and 0.8 are presented. The red circles represent the −log_10_(*P*-value) value assumed as following the χ^2^_1_ distribution, and the blue triangles represent the −log_10_(*P*-value) value assumed as following the 50:50 mixture (one component mixture is a peak at 0 and the other is a χ^2^_1_) distribution. The black line represents where the dots are expected to fall under the null hypothesis of no association. The plots show that the 50:50 mixture is more appropriate, and also reflect the fact that our simulations generated appropriate phenotypes under the null hypothesis of no phenotype-window correlation.

Figure S3 **The power to achieve 5% genome-wide significance for 100-SNP windows: the case of genome heritability 0.2 and 0.8**. The powers in the simulation study for genome heritability 0.2 and 0.8 were calculated by regional heritability mapping (RHM) with four different window sizes (100 SNPs as win100, 50 SNPs as win50, 20 SNPs as win20, and 10 SNPs as win10), and two single-SNP GWAS methods (GRAMMAR and GEMMA) in the different situations. The number of QTL is on the x-axis, and the power to detect QTL is on the y-axis. The results for genome heritability 0.2 are shown in **(A)** and 0.8 in **(B)**. The parameters considered in this simulation are QTL heritability (0.05 or 0.025) and MAF (low or high) in each genome heritability.

Figure S4 **The estimated regional heritability for 100-SNP windows: the case of genome heritability 0.2 and 0.8**. The regional heritabilities in the simulation study for genome heritability 0.2 and 0.8 were estimated by regional heritability mapping (RHM) with four different window sizes (100 SNPs as win100, 50 SNPs as win50, 20 SNPs as win20, and 10 SNPs as win10), and two single-SNP GWAS methods (GRAMMAR and GEMMA) in the different situations. The number of QTL is on the x-axis, and the estimated regional heritability is on the y-axis. The results for genome heritability 0.2 are shown in **(A)** and 0.8 in **(B)**. The parameters considered in this simulation are QTL heritability (0.05 or 0.025) and MAF (low or high) in each genome heritability.

Figure S5 **The power to achieve 5% genome-wide significance in the gene region: the case of genome heritability 0.2 and 0.8**. The powers in the simulation study for genome heritability 0.2 and 0.8 were calculated by regional heritability mapping with window size 10 (RHM), single-SNP GWAS (GEMMA), and three gene-based association approaches (VEGAS, CCA, and SKAT) in the different situations. The number of QTL is on the x-axis, and the power to detect QTL is on the y-axis. The results for genome heritability 0.2 are shown in **(A)** and 0.8 in **(B)**. The parameters considered in this simulation are QTL heritability (0.05 or 0.025) and MAF (low or high) in each genome heritability.

Figure S6 **Quantile–quantile plots for genome-wide association scan for three eye traits**. Quantile-quantile plots of 272,315 SNPs in the genome-wide association scan were shown for Axial Length, Central Corneal Thickness, and Spherical Equivalent Refraction by single-SNP GEMMA analysis. The red circles represent the observed statistics, and the black line represents where the dots are expected to fall under the null hypothesis of no association. The plots show that this method successfully accounts for population stratification.

Figure S7 **Genome-wide plots of −log_10_ (*P*-values) for an association with three eye traits**. Manhattan plots for Axial Length, Central Corneal Thickness, and Spherical Equivalent Refraction analyses by single-SNP GEMMA are shown. The genomic position is represented along the x-axis (chromosome number is indicated at the bottom of the plot). The −log_10_(*P*-value) is on the y-axis. The red dotted horizontal line is drawn at the 5% genome-wide significance. The significant threshold of genome-wide significance at 5% by Bonferroni correction was *P*-value = 1.8 × 10^−7^. For Central Corneal Thickness, there were two significant SNPs which were reported by Lu et al. (2013).

Figure S8 **Regional association plots for three eye traits near the significant region by regional heritability mapping (RHM) with window size 10 (win10)**. The results of regional association signals [higher −log_10_(*P*-value) > 5.0] are shown for Axial Length, Central Corneal Thickness, and Spherical Equivalent Refraction by RHM with win10. Plots were generated using LocusZoom (Pruim et al., 2010), and the color of each dot represents the SNP's linkage disequilibrium *r*^2^ in the HapMap Phase II CEU with the labeled SNP (1st SNP within win10 with the lowest *P*-value) plotted as a purple diamond. The blue bars show the recombination rate based on HapMap phase II CEU population, and the bottom panels illustrate the locations of known genes.

Table S1 **Descriptive statistics for Axial Length, Central Corneal Thickness, and Spherical Equivalent Refraction**.

Table S2
**SNP number and the average distance between SNPs by chromosome**.

Table S3 **Estimated genome heritability for Regional heritability mapping (RHM) in the high_info group of the simulation study**.
