# Supplementary figures and images for: The power of regional heritability analysis for rare and common variant detection: simulations and application to eye biometrical traits
Source: Front Genet. 2013 Nov 19;4:232. doi: 10.3389/fgene.2013.00232 (PMC3832942; doi:10.3389/fgene.2013.00232)

A

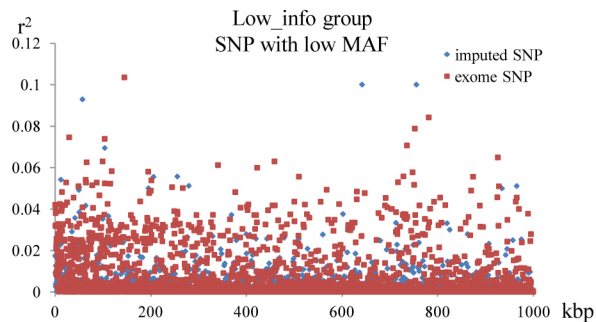

B

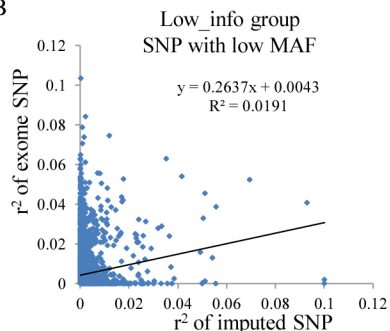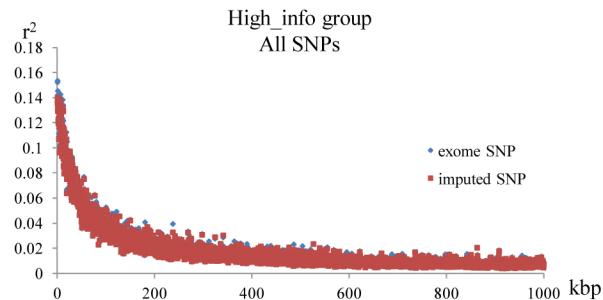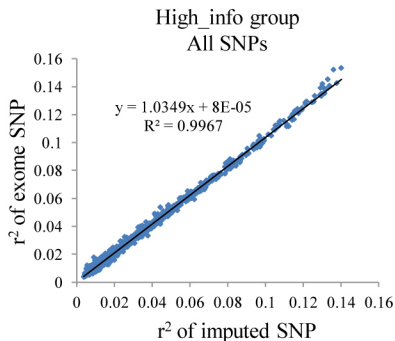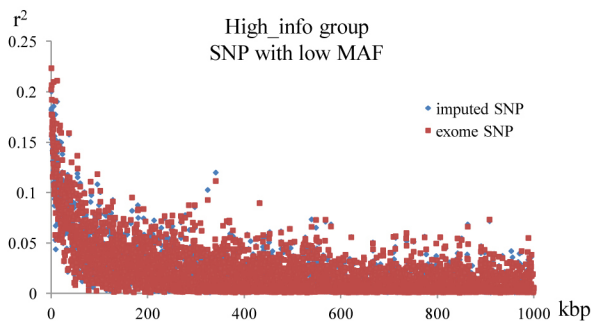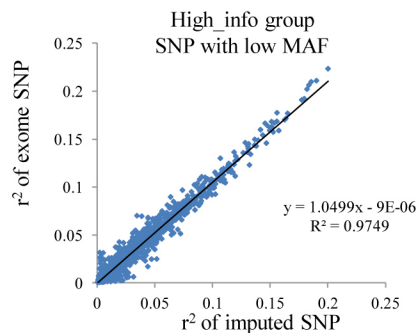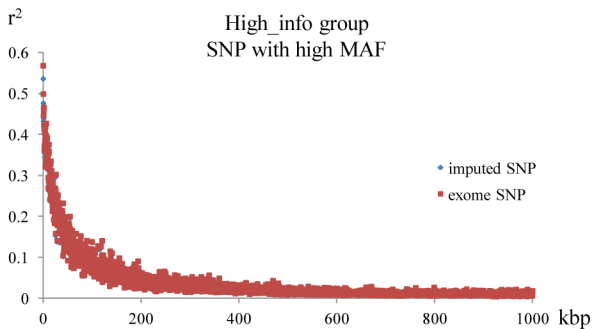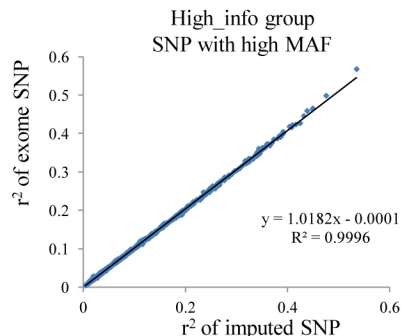

Supplement: Figure S1 — Average r2-value plotted against inter-marker distance and Correlation plot. To evaluate the quality of SNP imputation in this study, the difference of linkage disequilibrium (LD) between exome SNP and imputed SNP was investigated to evaluate whether the relationship between imputed SNPs is based on actual LD in this population or linkage equilibrium (LE). A total of 820 DNA samples from 898 Korcula samples were genotyped using the Illumina HumanExome-12v1 SNP array, that genotypes in excess of 250,000 exonic variants. These exome SNPs were then assessed by the exclusion criteria of minor allele frequency (MAF) <0.0005, call rate <0.98 and Hardy–Weinberg Equilibrium (HWE) < 1.0 × 10^−6^; SNPs included in Illumina CNV370 array were also excluded. A total of 7283 SNPs which were included in the low_info group and 25,313 SNPs in the high_info group were extracted from the exome array data. We estimated r2, a measure of LD, for all segregating pairs of SNPs less than 10 Mbp apart in each of these groups using the PLINK software (Purcell et al., 2007). Average r2-values for a given inter-marker distance, with markers distances grouped in 250 bp bins, were calculated in each autosome and plotted for each group. For the high_info group, r2-values for low MAF (MAF <0.10) and high MAF (MAF ≥ 0.10) SNPs were also calculated and plotted. The imputed SNPs for individuals with corresponding exome SNP data were extracted from the imputed SNP data, and r2-values were calculated as above. The correlation of r2-values obtained from genotyped SNPs (i.e., exome array data) and imputed SNPs was also estimated. The average r2-value was plotted against inter-SNP distance for exome SNPs and imputed SNPs in (A), and the correlation of r2-value between exome SNPs and imputed SNPs was also plotted in (B). In the high_info group, 12,636 SNPs with low MAF and 12,677 SNPs with high MAF were also used to calculate r2-values separately and then plotted. In the low_info group, there was no relations [file Presentation1.PDF]

A

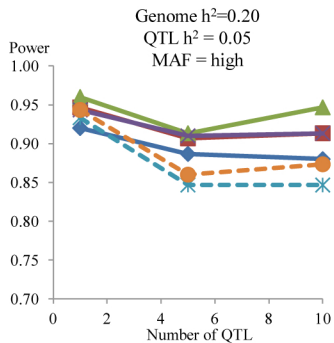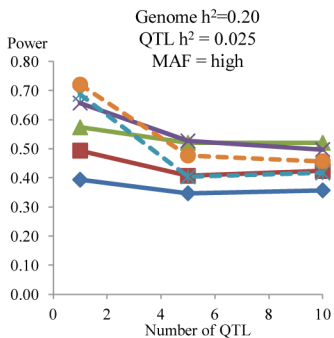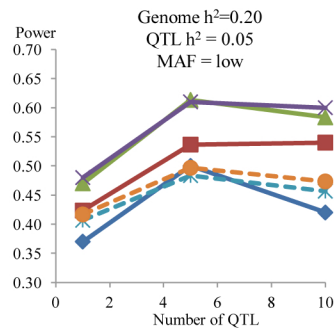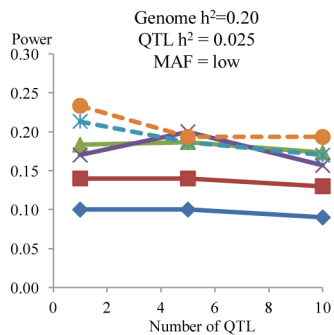

B

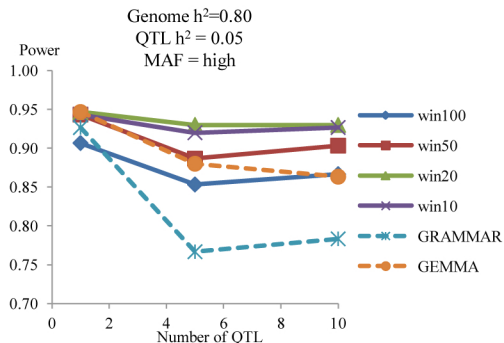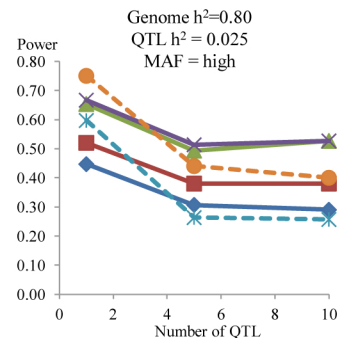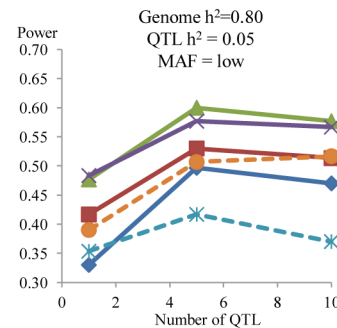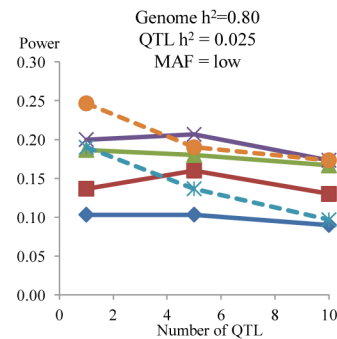

Supplement: Figure S3 — The power to achieve 5% genome-wide significance for 100-SNP windows: the case of genome heritability 0.2 and 0.8. The powers in the simulation study for genome heritability 0.2 and 0.8 were calculated by regional heritability mapping (RHM) with four different window sizes (100 SNPs as win100, 50 SNPs as win50, 20 SNPs as win20, and 10 SNPs as win10), and two single-SNP GWAS methods (GRAMMAR and GEMMA) in the different situations. The number of QTL is on the x-axis, and the power to detect QTL is on the y-axis. The results for genome heritability 0.2 are shown in (A) and 0.8 in (B). The parameters considered in this simulation are QTL heritability (0.05 or 0.025) and MAF (low or high) in each genome heritability. [file Presentation3.PDF]

A

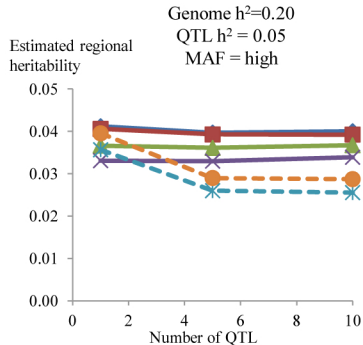

B

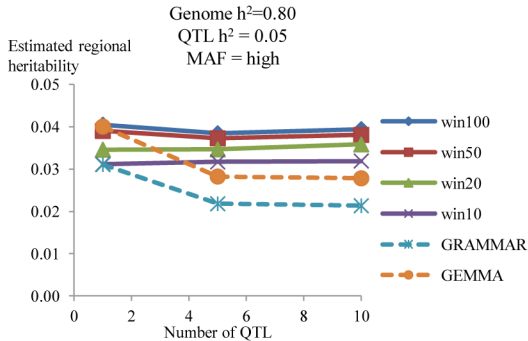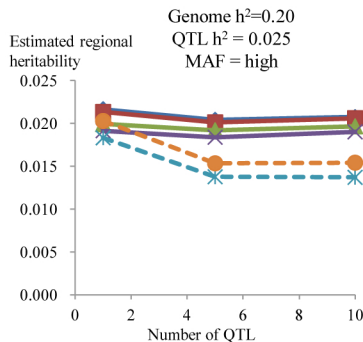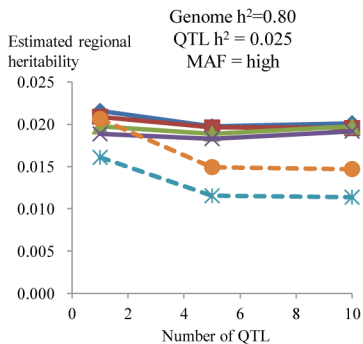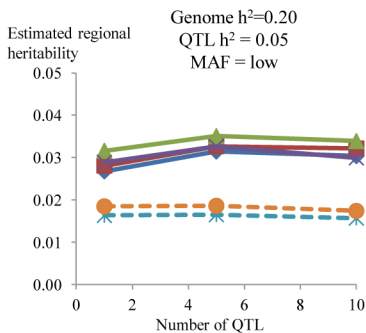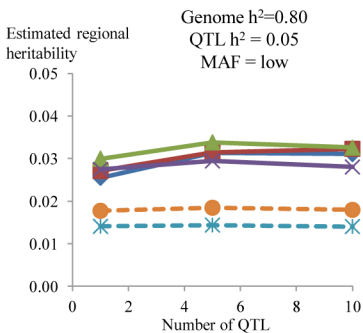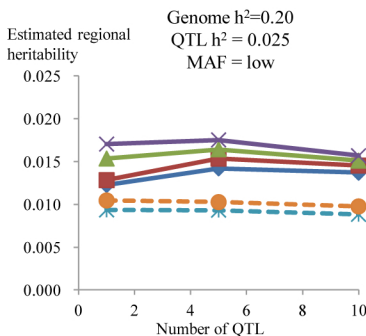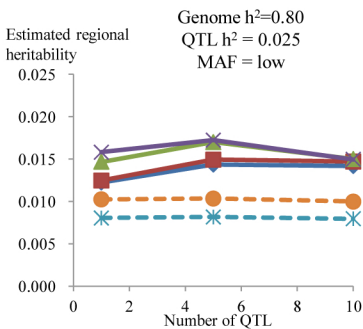

Supplement: Figure S4 — The estimated regional heritability for 100-SNP windows: the case of genome heritability 0.2 and 0.8. The regional heritabilities in the simulation study for genome heritability 0.2 and 0.8 were estimated by regional heritability mapping (RHM) with four different window sizes (100 SNPs as win100, 50 SNPs as win50, 20 SNPs as win20, and 10 SNPs as win10), and two single-SNP GWAS methods (GRAMMAR and GEMMA) in the different situations. The number of QTL is on the x-axis, and the estimated regional heritability is on the y-axis. The results for genome heritability 0.2 are shown in (A) and 0.8 in (B). The parameters considered in this simulation are QTL heritability (0.05 or 0.025) and MAF (low or high) in each genome heritability. [file Presentation4.PDF]

A

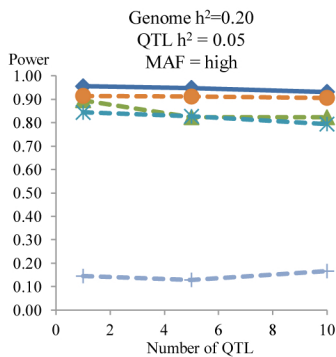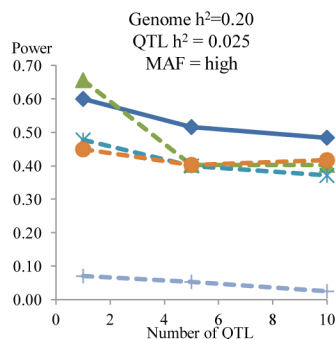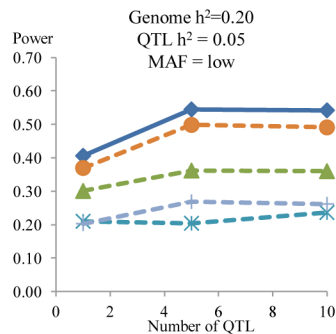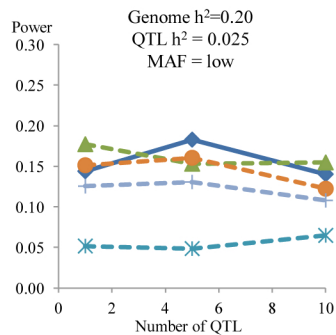

B

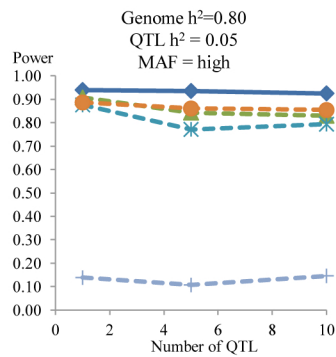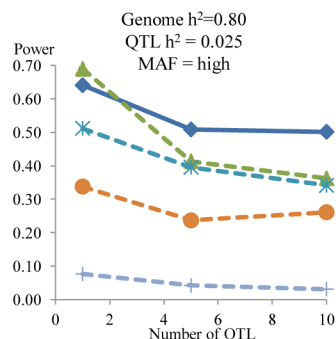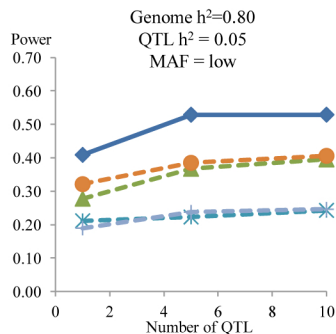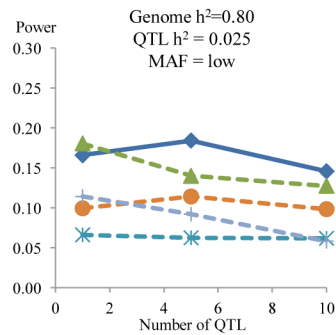

—◆— RHM    —▲— GEMMA  
—\*— VEGAS    —●— CCA  
—+— SKAT

Supplement: Figure S5 — The power to achieve 5% genome-wide significance in the gene region: the case of genome heritability 0.2 and 0.8. The powers in the simulation study for genome heritability 0.2 and 0.8 were calculated by regional heritability mapping with window size 10 (RHM), single-SNP GWAS (GEMMA), and three gene-based association approaches (VEGAS, CCA, and SKAT) in the different situations. The number of QTL is on the x-axis, and the power to detect QTL is on the y-axis. The results for genome heritability 0.2 are shown in (A) and 0.8 in (B). The parameters considered in this simulation are QTL heritability (0.05 or 0.025) and MAF (low or high) in each genome heritability. [file Presentation5.PDF]

Axial Length

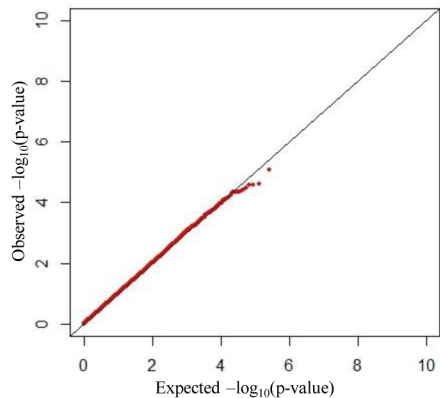

Central Corneal Thickness

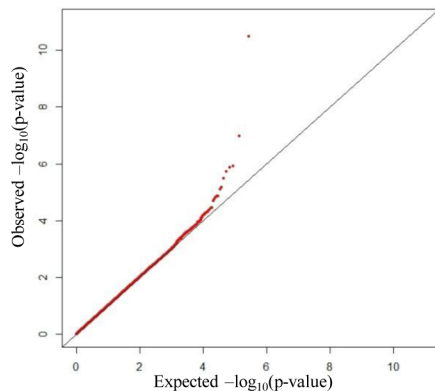

Spherical Equivalent Refraction

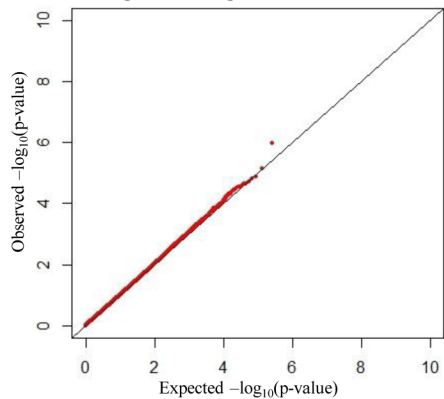

Supplement: Figure S6 — Quantile–quantile plots for genome-wide association scan for three eye traits. Quantile-quantile plots of 272,315 SNPs in the genome-wide association scan were shown for Axial Length, Central Corneal Thickness, and Spherical Equivalent Refraction by single-SNP GEMMA analysis. The red circles represent the observed statistics, and the black line represents where the dots are expected to fall under the null hypothesis of no association. The plots show that this method successfully accounts for population stratification. [file Presentation6.PDF]

## Axial Length

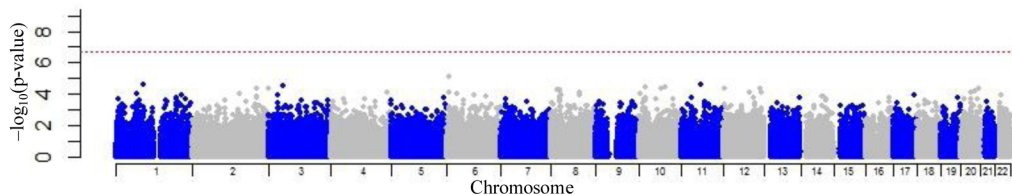

## Central Corneal Thickness

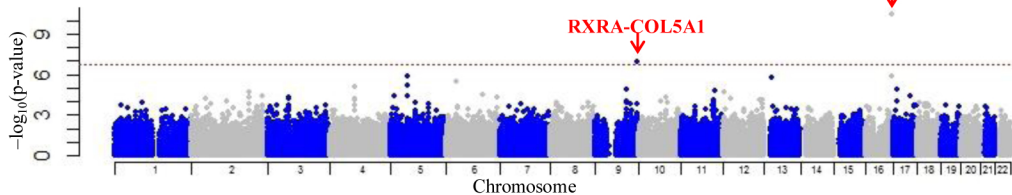

## Spherical Equivalent Refraction

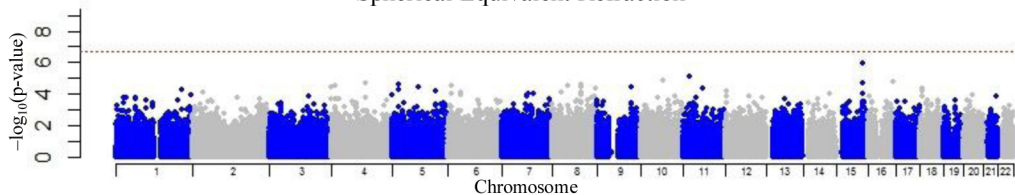

Supplement: Figure S7 — Genome-wide plots of −log10 (P-values) for an association with three eye traits. Manhattan plots for Axial Length, Central Corneal Thickness, and Spherical Equivalent Refraction analyses by single-SNP GEMMA are shown. The genomic position is represented along the x-axis (chromosome number is indicated at the bottom of the plot). The −log10(P-value) is on the y-axis. The red dotted horizontal line is drawn at the 5% genome-wide significance. The significant threshold of genome-wide significance at 5% by Bonferroni correction was P-value = 1.8 × 10^−7^. For Central Corneal Thickness, there were two significant SNPs which were reported by Lu et al. (2013). [file Presentation7.PDF]
