# Supplementary material for: The power of regional heritability analysis for rare and common variant detection: simulations and application to eye biometrical traits
Source: Front Genet. 2013 Nov 19;4:232. doi: 10.3389/fgene.2013.00232 (PMC3832942; doi:10.3389/fgene.2013.00232)
Supplement: Figure S2 — Quantile-quantile plot of the P-values for Regional Heritability Mapping (RHM) with 100-SNP-windows. For each value of simulated genome heritability (0.20, 0.40, and 0.8), the phenotype was generated, and then genome heritability was estimated by using model (2). The estimated genome heritabilities were 0.20 ± 0.07, 0.40 ± 0.07, and 0.80 ± 0.06 for the simulated values of 0.20, 0.40, and 0.80, respectively. We then performed RHM analyses using 100-SNP window (win100) using these phenotypes, to obtain empirically a distribution of test statistics under the null hypothesis. For each genome heritability, a quantile-quantile plot of the P-values of the RHM analyses with win100 assuming that they follow either a 50:50 mixture distribution of a χ21 and a pick at 0 or a χ21 distribution are shown. Results of analysis of generated phenotypes with genome heritability = 0.2, 0.4, and 0.8 are presented. The red circles represent the −log10(P-value) value assumed as following the χ21 distribution, and the blue triangles represent the −log10(P-value) value assumed as following the 50:50 mixture (one component mixture is a peak at 0 and the other is a χ21) distribution. The black line represents where the dots are expected to fall under the null hypothesis of no association. The plots show that the 50:50 mixture is more appropriate, and also reflect the fact that our simulations generated appropriate phenotypes under the null hypothesis of no phenotype-window correlation. [file Presentation2.PDF]

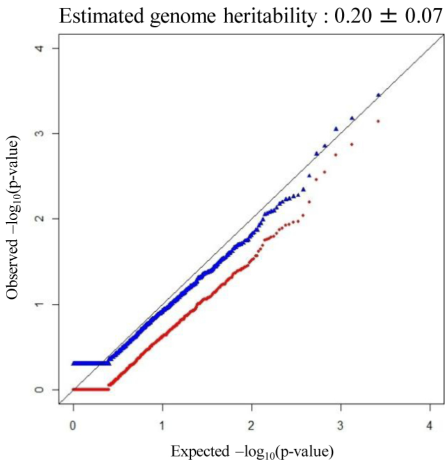
Estimated genome heritability : 0.20 ± 0.07
Observed −log10(p-value)
Expected −log10(p-value)
0
1
2
3
4

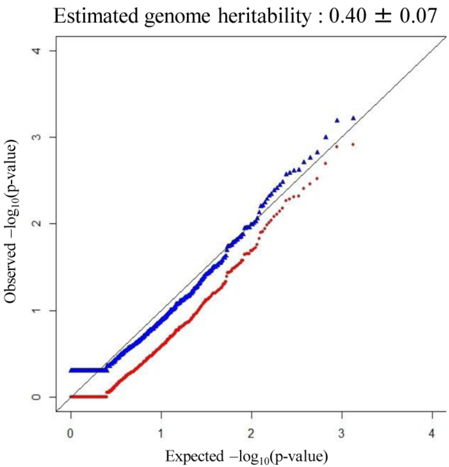
Estimated genome heritability : 0.40 ± 0.07
Observed −log10(p-value)
Expected −log10(p-value)
0
1
2
3
4

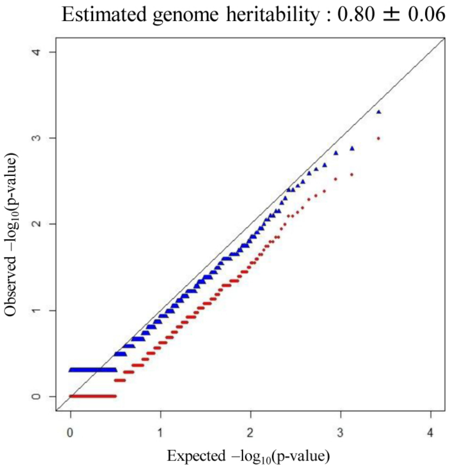
Estimated genome heritability : 0.80 ± 0.06
Observed −log10(p-value)
Expected −log10(p-value)
0
1
2
3
4
