# Supplementary material for: The power of regional heritability analysis for rare and common variant detection: simulations and application to eye biometrical traits
Source: Front Genet. 2013 Nov 19;4:232. doi: 10.3389/fgene.2013.00232 (PMC3832942; doi:10.3389/fgene.2013.00232)
Supplement: Figure S8 — Regional association plots for three eye traits near the significant region by regional heritability mapping (RHM) with window size 10 (win10). The results of regional association signals [higher −log10(P-value) > 5.0] are shown for Axial Length, Central Corneal Thickness, and Spherical Equivalent Refraction by RHM with win10. Plots were generated using LocusZoom (Pruim et al., 2010), and the color of each dot represents the SNP's linkage disequilibrium r2 in the HapMap Phase II CEU with the labeled SNP (1st SNP within win10 with the lowest P-value) plotted as a purple diamond. The blue bars show the recombination rate based on HapMap phase II CEU population, and the bottom panels illustrate the locations of known genes. [file Presentation8.PDF]

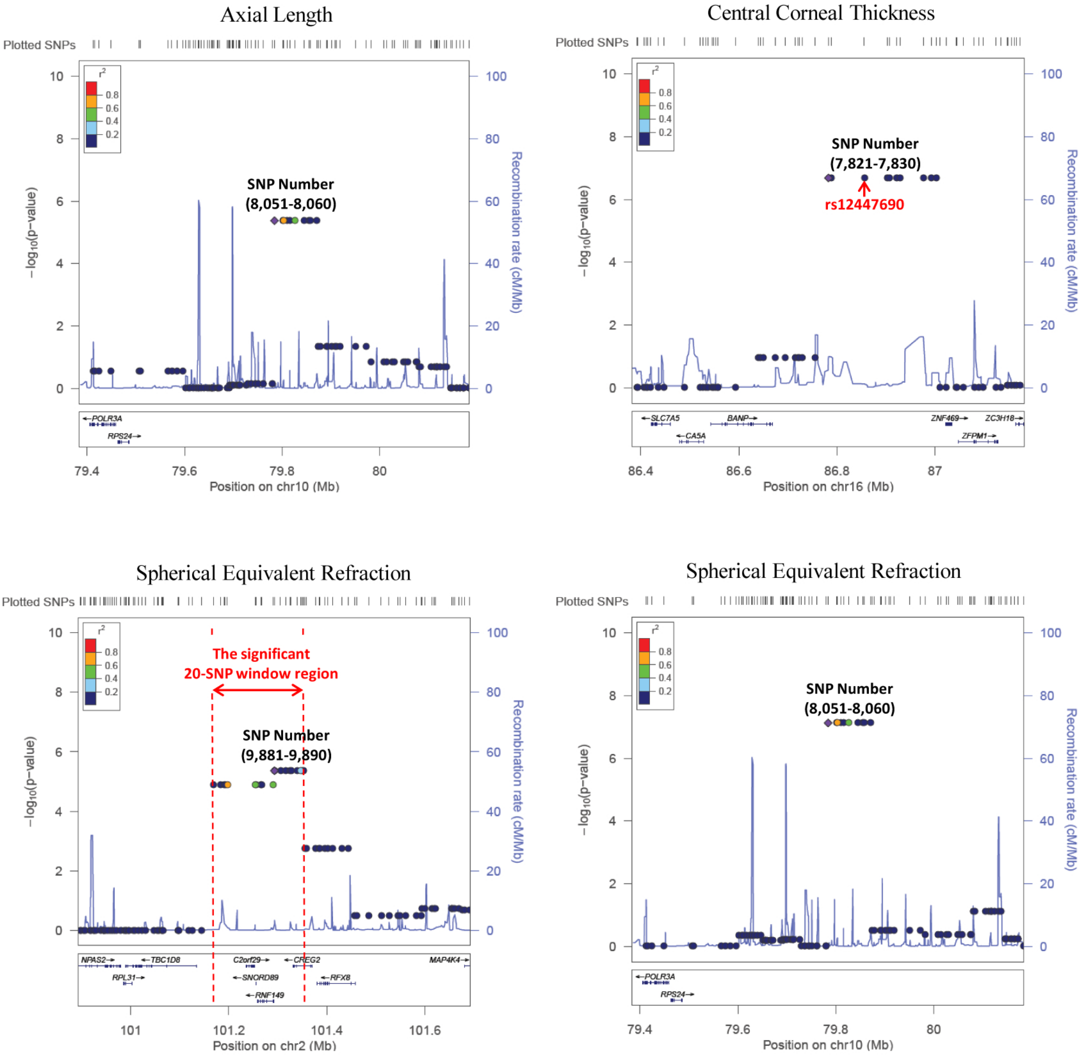
Axial Length
Plotted SNPs
SNP Number
(8,051-8,060)
Position on chr10 (Mb)
−log10(p−value)
Recombination rate (cM/Mb)
POLR3A
RPS24
Central Corneal Thickness
SNP Number
(7,821-7,830)
rs12447690
Position on chr16 (Mb)
SLC7A5
CA5A
BANP
ZNF469
ZFPM1
ZC3H18
Spherical Equivalent Refraction
The significant
20-SNP window region
SNP Number
(9,881-9,890)
Position on chr2 (Mb)
NPAS2
TBC1D8
RPL31
C2orf29
SNORD89
RNF149
CREG2
RFX8
MAP4K4
Spherical Equivalent Refraction
SNP Number
(8,051-8,060)
Position on chr10 (Mb)
POLR3A
RPS24
