# Supplementary material for: The power of regional heritability analysis for rare and common variant detection: simulations and application to eye biometrical traits
Source: Front Genet. 2013 Nov 19;4:232. doi: 10.3389/fgene.2013.00232 (PMC3832942; doi:10.3389/fgene.2013.00232)
Supplement: Table S1 — Descriptive statistics for Axial Length, Central Corneal Thickness, and Spherical Equivalent Refraction. [file DataSheet1.PDF]

Supplementary Table 1. Descriptive statistics for Axial Length, Central Corneal Thickness, and Spherical Equivalent Refraction.

Traits	Population	Genotype N	Phenotype N	Average	SD	Heritability
Axial Length (AL)	Vis	924	558	23.12	1.03	
	Korcula	898	829	23.22	1.12	
	Orkney	889	504	23.72	1.10	
	Split	499	354	23.42	1.02	
	Total	3,210	2,245	23.34	1.10	0.50
Central Corneal Thickness (CCT)	Vis	924	576	560.9	34.5	
	Korcula	898	860	558.1	36.8	
	Orkney	889	476	537.6	33.0	
	Split	499	349	560.4	35.9	
	Total	3,210	2,261	554.8	36.4	0.83
Spherical Equivalent Refraction (SER)	Vis	924	542	-0.21	2.03	
	Korcula	898	836	-0.24	1.83	
	Orkney	889	508	0.00	2.14	
	Split	499	365	-1.83	1.87	
	Total	3,210	2,251	-0.44	2.05	0.43

Heritabilities were estimated using the mixed model equation (2) accounting for non-genetic effect.

Supplementary Table 2. SNP number and the average distance between SNPs by chromosome.

Chromosome	Number	Average distance (kbp)
1	20,931	11.7
2	22,494	10.8
3	19,147	10.4
4	16,564	11.5
5	16,907	10.7
6	18,394	9.3
7	14,771	10.7
8	15,973	9.1
9	14,079	9.8
10	13,800	9.8
11	12,945	10.4
12	13,354	9.9
13	10,146	9.5
14	8,774	9.9
15	7,868	10.4
16	7,987	11.1
17	7,396	10.6
18	9,204	8.3
19	5,084	12.5
20	6,859	9.1
21	4,847	6.9
22	4,791	7.1
Total	272,315	10.2

Supplementary Table 3. Estimated genome heritability for Regional heritability mapping (RHM) in the high_info group of the simulation study.

MAF	Phenotype heritability	QTL heritability	Number of QTL	Null	
				Mean	SD
High	0.2	0.05	1	0.195	0.054
			5	0.190	0.056
			10	0.192	0.053
		0.025	1	0.197	0.040
			5	0.194	0.042
			10	0.196	0.040
	0.4	0.05	1	0.398	0.035
			5	0.398	0.034
			10	0.396	0.034
		0.025	1	0.400	0.025
			5	0.400	0.025
			10	0.399	0.024
	0.8	0.05	1	0.797	0.020
			5	0.795	0.022
			10	0.795	0.021
		0.025	1	0.799	0.015
			5	0.798	0.015
			10	0.797	0.014
Low	0.2	0.05	1	0.189	0.058
			5	0.188	0.057
			10	0.187	0.055
		0.025	1	0.193	0.043
			5	0.191	0.041
			10	0.192	0.042
	0.4	0.05	1	0.393	0.035
			5	0.391	0.035
			10	0.392	0.033
		0.025	1	0.397	0.024
			5	0.396	0.025
			10	0.397	0.025
	0.8	0.05	1	0.788	0.021
			5	0.789	0.021
			10	0.789	0.021
		0.025	1	0.794	0.014
			5	0.794	0.015

	10	0.794	0.014

MAF	Phenotype heritability	QTL heritability	number of QTL	RHM[1] win100 Mean	RHM[1] win100 SD	RHM[1] win50 Mean	RHM[1] win50 SD
High	0.2	0.05	1	0.144	0.050	0.145	0.051
			5	0.145	0.050	0.145	0.050
			10	0.145	0.050	0.145	0.049
		0.025	1	0.171	0.038	0.171	0.038
			5	0.172	0.039	0.171	0.038
			10	0.172	0.038	0.172	0.038
	0.4	0.05	1	0.355	0.031	0.356	0.030
			5	0.356	0.031	0.358	0.030
			10	0.356	0.031	0.357	0.030
		0.025	1	0.378	0.024	0.379	0.023
			5	0.379	0.024	0.379	0.023
			10	0.378	0.023	0.379	0.023
	0.8	0.05	1	0.755	0.019	0.756	0.019
			5	0.756	0.020	0.756	0.020
			10	0.755	0.020	0.755	0.021
		0.025	1	0.777	0.015	0.777	0.014
			5	0.778	0.016	0.777	0.015
			10	0.777	0.015	0.777	0.015
Low	0.2	0.05	1	0.163	0.056	0.163	0.057
			5	0.158	0.053	0.158	0.053
			10	0.158	0.051	0.157	0.051
		0.025	1	0.181	0.042	0.181	0.042
			5	0.177	0.039	0.177	0.040
			10	0.178	0.040	0.178	0.040
	0.4	0.05	1	0.370	0.035	0.369	0.035
			5	0.366	0.034	0.365	0.033
			10	0.364	0.033	0.363	0.033
		0.025	1	0.386	0.025	0.386	0.024
			5	0.385	0.025	0.385	0.024
			10	0.384	0.025	0.383	0.024
	0.8	0.05	1	0.764	0.025	0.763	0.027
			5	0.760	0.023	0.759	0.024
			10	0.759	0.022	0.758	0.022
		0.025	1	0.782	0.016	0.782	0.017
			5	0.780	0.016	0.780	0.016
			10	0.781	0.015	0.780	0.016

MAF	Phenotype heritability	QTL heritability	number of QTL	RHM[1] win20 Mean	RHM[1] win20 SD	RHM[1] win10 mean	RHM[1] win10 SD
High	0.2	0.05	1	0.146	0.050	0.148	0.050
			5	0.147	0.049	0.149	0.050
			10	0.146	0.049	0.148	0.050
		0.025	1	0.172	0.038	0.172	0.037
			5	0.171	0.037	0.172	0.037
			10	0.172	0.037	0.173	0.037
	0.4	0.05	1	0.357	0.030	0.359	0.031
			5	0.359	0.030	0.361	0.030
			10	0.358	0.029	0.360	0.030
		0.025	1	0.379	0.022	0.379	0.022
			5	0.379	0.022	0.380	0.023
			10	0.379	0.022	0.380	0.023
	0.8	0.05	1	0.760	0.018	0.762	0.018
			5	0.758	0.018	0.760	0.019
			10	0.757	0.020	0.759	0.020
		0.025	1	0.778	0.013	0.779	0.013
			5	0.778	0.014	0.778	0.014
			10	0.777	0.015	0.777	0.015
Low	0.2	0.05	1	0.162	0.057	0.163	0.058
			5	0.158	0.052	0.160	0.053
			10	0.157	0.052	0.159	0.052
		0.025	1	0.180	0.042	0.179	0.042
			5	0.177	0.040	0.177	0.039
			10	0.178	0.040	0.178	0.040
	0.4	0.05	1	0.369	0.034	0.369	0.034
			5	0.366	0.033	0.367	0.035
			10	0.364	0.033	0.365	0.033
		0.025	1	0.386	0.024	0.386	0.023
			5	0.384	0.024	0.384	0.024
			10	0.384	0.024	0.384	0.025
	0.8	0.05	1	0.760	0.030	0.762	0.029
			5	0.757	0.025	0.760	0.025
			10	0.757	0.023	0.761	0.023
		0.025	1	0.780	0.018	0.779	0.019

5	0.778	0.017	0.777	0.018
10	0.780	0.016	0.779	0.016

Null: genome heritability estimated by model (2), RHM: genome heritability estimated by model (1).
